# Efficacy and Safety of Antiretroviral Therapy Initiated One Week after Tuberculosis Therapy in Patients with CD4 Counts < 200 Cells/μL: TB-HAART Study, a Randomized Clinical Trial

**DOI:** 10.1371/journal.pone.0122587

**Published:** 2015-05-12

**Authors:** Wondwossen Amogne, Getachew Aderaye, Abiy Habtewold, Getnet Yimer, Eyasu Makonnen, Alemayhu Worku, Anders Sonnerborg, Eleni Aklillu, Lars Lindquist

**Affiliations:** 1 Department of Medicine, Division of Infectious Diseases, Karolinska Institute at Karolinska University Hospital Huddinge, Stockholm, Sweden; 2 Department of Internal Medicine, School of Medicine, Addis Ababa University, Addis Ababa, Ethiopia; 3 Division of Clinical Pharmacology, Department of Laboratory Medicine, Karolinska University Hospital, Huddinge C1: 68, Karolinska Institute, Stockholm, Sweden; 4 Department of Pharmacology, School of Medicine, Addis Ababa University, Addis Ababa, Ethiopia; 5 School of Public Health, Addis Ababa University, Addis Ababa, Ethiopia; University of Ottawa, CANADA

## Abstract

**Background:**

Given the high death rate the first two months of tuberculosis (TB) therapy in HIV patients, it is critical defining the optimal time to initiate combination antiretroviral therapy (cART).

**Methods:**

A randomized, open-label, clinical trial comparing efficacy and safety of efavirenz-based cART initiated one week, four weeks, and eight weeks after TB therapy in patients with baseline CD4 count < 200 cells/μL was conducted. The primary endpoint was all-cause mortality rate at 48 weeks. The secondary endpoints were hepatotoxicity-requiring interruption of TB therapy, TB-associated immune reconstitution inflammatory syndrome, new AIDS defining illnesses, CD4 counts, HIV RNA levels, and AFB smear conversion rates. All analyses were intention-to-treat.

**Results:**

We studied 478 patients with median CD4 count of 73 cells/μL and 5.2 logs HIV RNA randomized to week one (n = 163), week four (n = 160), and week eight (n = 155). Sixty-four deaths (13.4%) occurred in 339.2 person-years. All-cause mortality rates at 48 weeks were 25 per 100 person-years in week one, 18 per 100 person-years in week four and 15 per 100 person-years in week eight (P = 0.2 by the log-rank test). All-cause mortality incidence rate ratios in subgroups with CD4 count below 50 cells/μL versus above were 2.8 in week one (95% CI 1.2–6.7), 3.1 in week four (95% CI 1.2–8.6) and 5.1 in week eight (95% CI 1.8–16). Serum albumin < 3gms/dL (adjusted HR, aHR = 2.3) and CD4 < 50 cells/μL (aHR = 2.7) were independent predictors of mortality. Compared with similar subgroups from weeks four and eight, first-line TB treatment interruption was high in week one deaths (P = 0.03) and in the CD4 subgroup <50 cells/μL (P = 0.02).

**Conclusions:**

Antiretroviral therapy one week after TB therapy doesn’t improve overall survival. Despite increased mortality with CD4 < 50 cells/μL, we recommend cART later than the first week of TB therapy to avoid serious hepatotoxicity and treatment interruption.

**Trial Registration:**

ClinicalTrials.gov NCT 01315301

## Introduction

Tuberculosis (TB) is the most frequent cause of death in HIV infected patients. People living with HIV are 27–32 times more likely to develop TB than HIV-negatives. In 2012, 30% of the 1.1 million new TB cases in HIV-positives died. Of the new cases, 75% were in Africa [1]. One third of the TB/HIV coinfection in Africa occur at CD4 count < 200 cells/μL [2,3]. The incidence rate of TB among South Africans with CD4 < 200 cells/μL was 40 per 100 person years [4]. Both the incidence rate and prognosis of TB in high burden areas are influenced by low baseline CD4 counts [5,6,7]. TB case fatality ratios (CFR) in African and Asian patients not receiving combination antiretroviral therapy (cART) or in whom it was deferred for 8 weeks varied from 16–35% [8,9,10]. In settings where HIV prevalence is high, the CFR was highest in the first two months of TB therapy suggesting cART should begin early [9]. Early initiation of cART, within the first eight weeks of TB treatment or within two weeks for CD4 < 50cells/μL is recommended [11,12,13]. Mortality risk was reduced by 56% with cART initiated while on TB treatment rather than at completion [14]. The optimal balance between the potential benefits and risks directs cART timing during TB therapy. The potential benefit of starting cART before two weeks is earlier immune restoration and improved survival. Whereas the potential risks are augmented toxicities, Tuberculosis Immune Reconstitution Inflammatory Syndrome (TB-IRIS), drug interactions and pill burden that complicate treatment outcome and patient care. Various studies showed no differences in incidence rates of AIDS- defining illnesses or death when cART initiated between two to eight weeks of TB therapy as compared with later except for sub-groups with CD4 count < 50 cells/μL[12,15,16,17]. Our study hypothesis is that initiating cART earlier than second week of TB therapy in patients with CD4 counts < 200 cells/ μL, will improve overall survival at 48 weeks.

We conducted a randomized, controlled trial of one week versus four and eight weeks initiation of cART in TB/HIV co infected patients with CD4 count < 200cells/ μL, to determine whether one week reduces the risk of death.

## Methods

### Study design and oversight

Multicenter, open-label, parallel, randomized clinical trial was conducted in Addis Ababa, Ethiopia from June 2, 2008 until April 22, 2011. The trial started after the Institutional Review Board of the School of Medicine at Addis Ababa University, the Ethiopian Science and Technology Agency as well as Food, Medicine and Health Care Administration and Control authority of Ethiopia approved the study protocol. The study was officially stopped after the last scheduled follow up visit. It was registered at clinical trials.gov after recruitment started. There are no other clinical trials related with this study. Data Safety Monitoring Board supervised progress of the study. The protocol for this trial, supporting CONSORT checklist, and the data are available as supporting information; see S1 CONSORT Checklist, S1 Protocol, and S1 Data.

### Participants

The patients were recruited from four trial sites within Addis Ababa. The main trial site was Tikur Anbessa Specialized Referral Hospital and others were health centres. Eligible subjects were ambulatory with suspected or confirmed new TB diagnosis. They were 18 years or older with confirmed HIV infection (CD4 count < 200 cells/μL) and written informed consent obtained on enrolment. The diagnosis of TB was based on WHO criteria for the diagnosis of smear positive pulmonary TB (SP-PTB), smear negative pulmonary TB (SN-PTB) and extrapulmonary TB (EPTB) [18]. Miliary form of TB with pulmonary involvement (PTB) and EPTB manifested with PTB were classified as PTB. Components of the WHO criteria are sputum AFB result, chest X-ray findings, clinician’s decision to treat for TB and histological evidences.

Exclusion criteria were TB of the central nervous system, Karnofsky score < 40, serum alanine aminotransferase more than 3 times the upper limit of normal (ULN), hemoglobin < 8 gms/dL, previous antiretroviral therapy exposure and pregnancy.

### Interventions

The study subjects received first-line TB therapy with weight adjusted fixed dose combinations of rifampicin, isoniazid, ethambutol, and pyrazinamide for 2 months (intensive phase) and a subsequent fixed dose combination of isoniazid and rifampicin for 4 months (continuation phase). The study participants were randomized in to three groups. The first received cART one week (week one), the second four weeks (week four) and the third eight weeks (week eight) after TB therapy started. The median number of TB treatment days before cART was initiated defined the different study groups. The cART regimen included efavirenz (600 mg once daily) plus lamivudine, and study site physician selection of zidovudine, stavudine or tenofovir. Time to initiate cART was deferred in grade 3 and 4 hepatotoxicity events and while first-line TB therapy interrupted due to hepatotoxicity. All patients received cotrimoxazole 960 mgs once daily. The participants were assessed at enrolment (baseline), and subsequently in weeks 1, 2, 4, 6, 8, 12, 20, 24 and 48 weeks. At each visit complete blood count, serum levels of aspartate aminotransferase (AST), alanine aminotransferase (ALT), alkaline phosphatase (ALP), bilirubin (total and direct), urea and creatinine were measured. At 12 weeks intervals, CD4 count and HIV RNA levels were determined until the 24^th^ week, and then at 48^th^ week. Hepatitis B surface Antigen (HBsAg) and Hepatitis C Virus Antibody (HCV Ab) status were confirmed at baseline. Sputum for acid-fast bacilli (AFB) was examined with Ziehl-Neelsen stain when PTB was first diagnosed and at eighth week of TB therapy to evaluate smear conversion. For the diagnosis of TB-associated IRIS, a case definition for use in resource-limited settings was applied [19] where at least one major criterion or two minor clinical criteria were required. Hepatotoxicity was graded using the Division of AIDS Table for Grading of Severity of Adult and Pediatric Adverse events (“DAIDS AE grading table”), December 2004 version. First-line TB treatment was interrupted when ALT value was elevated more than three times the upper limit of normal (ULN) with symptoms and/or jaundice (total bilirubin >2xULN), or five times the ULN in the absence of symptoms according to the American Thoracic Society (ATS) official statement, 2006 [20]. Test reference values for the ULN were used (ALT 30 iu/L for females, 40 iu/L for males, ALP 306 iu/L and total bilirubin1.0 mg/dL). First-line TB treatment interruption was defined as any period in which fixed dose combination TB therapy was discontinued because of hepatotoxicity. During this time, TB treatment was modified to continue with streptomycin and ethambutol. Depending on its availability, moxifloxacin was added. The first-line TB drugs were reintroduced when serum ALT and the total bilirubin values dropped to ≤ 2xULN. The outcome of TB therapy was evaluated with standard 10 of international standards for tuberculosis care [21].

### Study hypothesis & end points

The primary hypothesis was initiation of cART one week after TB therapy reduces all-cause mortality rate at 48 weeks compared with week four as well as week eight. All-cause mortality rates from the three study groups are compared. The primary end point was all-cause mortality rate assessed 48 weeks after study entry. Cause of death was abstracted from medical records or from verbal autopsy data. It was classified AIDS-related if an AIDS- defining illness (category C disease listed by the CDC and WHO stage IV events), pneumonia or sepsis was listed as contributing cause. The secondary end points were: incidence rates of hepatotoxicity requiring interruption of first-line TB therapy, TB-IRIS and AIDS defining illnesses (ADIs), HIV-related outcomes (increase in the median CD4 counts and proportions of patients with undetectable HIV-1 RNA i.e. ≤ 400 copies/ mL) and TB-treatment outcome (AFB smear converted negative at eighth week).

### Sample size & statistical analysis

The estimated sample size was 450 patients (factoring in anticipated 20% loss). It has 80% power, alpha level of 0.05 to detect 50% reduction in mortality from predicted CFR of 25% when cART is started after the 8^th^ week of TB therapy. All statistical analyses were according to the intention-to-treat principles. The primary and secondary end points were analysed with Kaplan-Meier curves and log-rank test. The duration of time in the study was calculated as the time from TB treatment to death, lost to follow-up (LTF), or completion of the 48 weeks of the study which ever occurred first. For continuous outcome variables, multiple levels comparisons were made with one-way ANOVA. Cox proportional-hazards model was used to explore the probability of death before and after TB therapy adjusting for confounding variables and factors of interest. The factors which had P-value ≤ 0.1 in univariate analysis and of interest were included in the multivariate model. Pearson chi-square test was applied for secondary endpoint analysis. Assumptions for each analytical method were checked. SPSS for windows version 20 and STATA 11 software were used for the statistical analysis. P-values less than 0.05 were considered statistically significant.

### Randomization

Eligible participants were assigned to one of the three study weeks with simple randomization. Each site prepared separate randomization list. The random order of assignment was determined in advance by lots method.

## Results

### Study participants and recruitment

**Fig 1:**
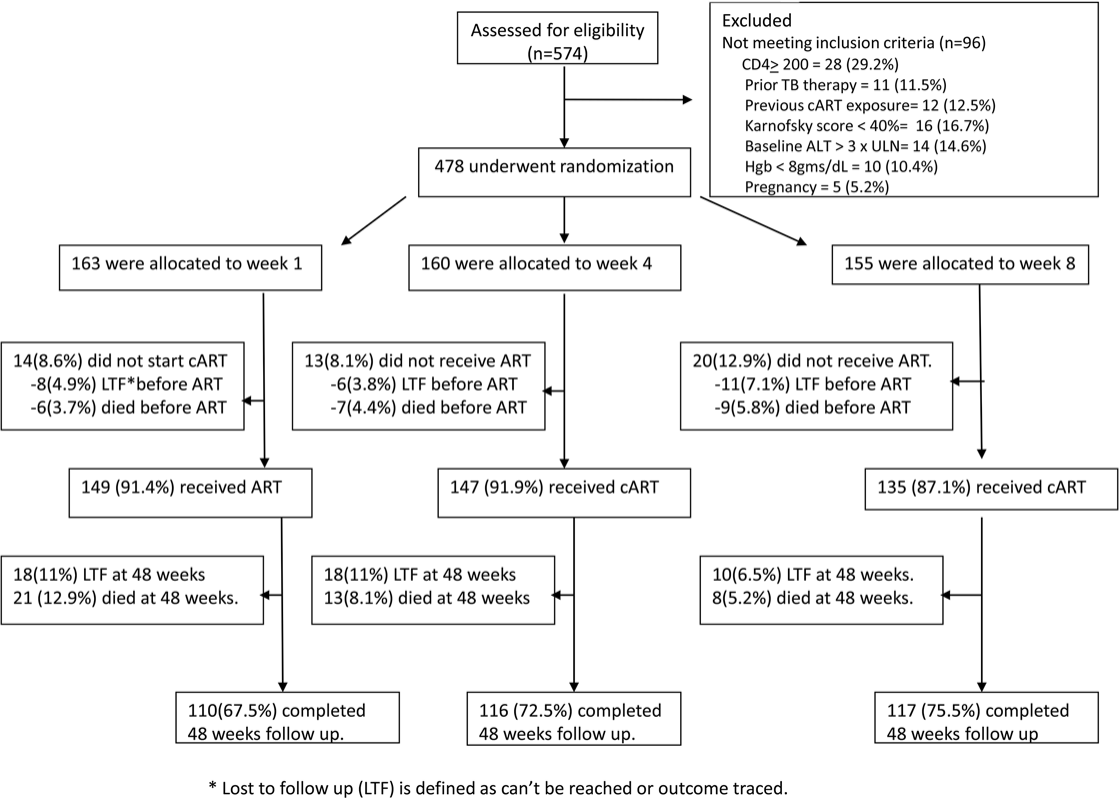
Consort diagram showing study participants enrolment and outcomes.

A total of 478 TB and HIV co-infected patients (233 women) were enrolled in the study: 163 were assigned to week one, 160 to week four and 155 to week eight. Patient enrolments and outcome characteristics are shown in Fig 1 and baseline study group characteristics in Table 1. Baseline median (IQR) values were: CD4 count 73 (44–110) cells/μL, HIV RNA level 5.2 (4.6–5.6) log copies/ mL and BMI 18.4(16.8–20.2) Kgs/m^2^. The TB cases were classified as SP-PTB (21%), SN-PTB (53%) and EPTB (26%). TB lymphadenitis accounted for 78% of the EPTB cases. The total follow up duration was 339.2 person-years and the median (IQR) follow up was for 11.2 months (4.7–11.2). Numbers of patients who completed the 48 weeks of the study were 110 (67.5%), 116 (72.5%) and 117 (75.5%) in weeks one, four and eight respectively.

**Table 1:** Patient characteristics at enrolment.

Variables	week one (163)	week four (160)	week eight (155)
Age, yrs (mean, sd)	37(10)	36(9)	35(10)
Male sex, no (%)	93(57%)	88(55%)	64(41%)
BMI, Kgs/m^2^ (mean, sd)	18.9 (2.9)	18.7(3.1)	18.6(3)
Type TB diagnosis (no, %)			
SP-PTB	29(17.8%)	28(17.5%)	42(27.1%)
SN-PTB	91(55.8%)	85(53.1%)	78(50.3%)
EPTB	43(26.4%)	47(29.4%)	35(22.6%)
CD4 cells/μL (median,IQR)	67(39–106)	71(45–121)	76(47–110)
CD4 < 50 cells/μL, no (%)	61(37.4%)	50(31.3%)	39(25.2%)
CD4 ≥ 50 cells/ μL, no (%)	102(62.6%)	110(68.7%)	116(74.8%)
Log HIV-RNA (mean, sd)	4.9(0.7)	5.1(0.8)	5.2(0.7)
Karnofsky score% (median,IQR)	90(70–100)	85(70–100)	90(80–100)
TB treatment days before cART(median, range)	7 (4–48)	28(21–45)	56(48–74)
HBsAg positive, no (%)	16(9.8%)	14(8.8%)	11(7.1%)
HCVAb positive, no (%)	4(2.5%)	4(2.5%)	0
Serum albumin, gms/dL (mean, sd)	3.3 (0.7)	3.5(0.8)	3.3(0.7)
cART regimen: efavirenz/lamivudine and			
not started	13(8%)	14(8.9%)	18(11.6%)
stavudine	41(25.2%)	61(38.1%)	34(21.9%)
zidovudine	47(28.8%)	41(25.6%)	49(31.6%)
tenofovir	62(38%)	44(27.5%)	54(34.8%)

### Initiation of ART

The median (range) numbers of days before cART initiation were 7days (4–48), 28 days (21–45) and 56 days (48–74) in weeks one, four and eight respectively.

### Primary end point

**Fig 2:**
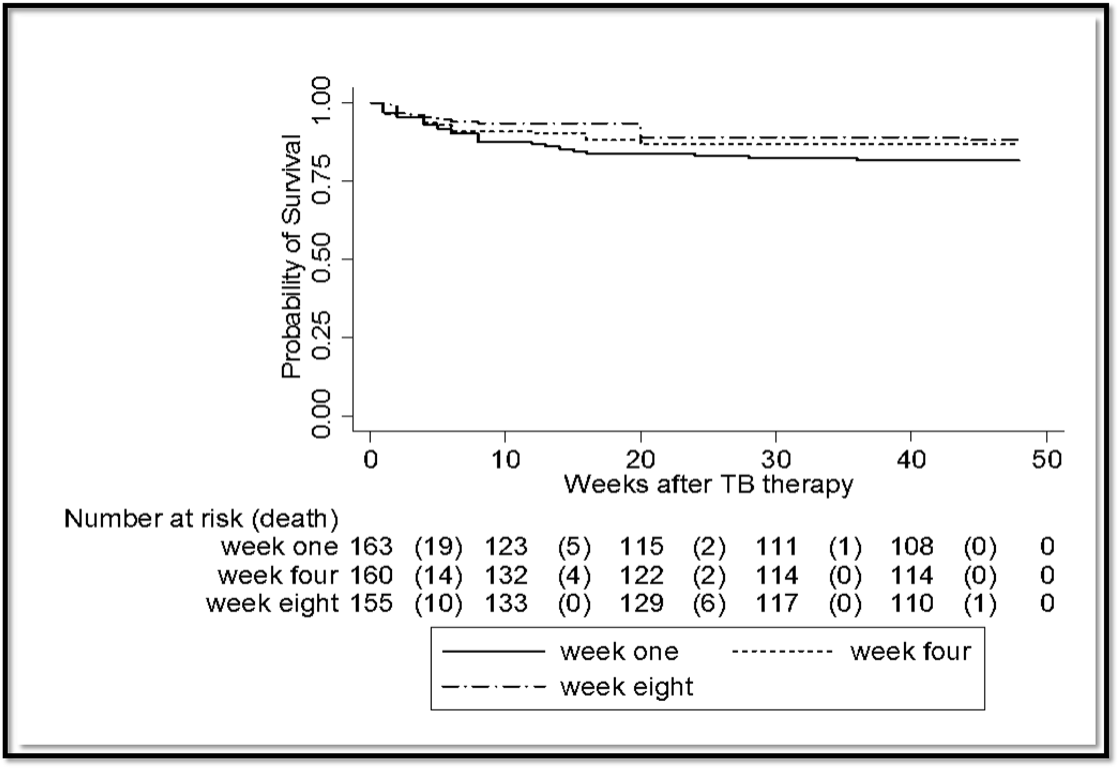
Comparison of all-cause mortality rates with Kaplan-Meier Survival Estimates according to the different study weeks, X^2^ log-rank = 2.9, df = 2, P = 0.2.

There were a total of 64 deaths (Case fatality ratio, CFR = 13.4%) in 339.2 person-years of follow up. Twenty-seven cases were in week one, 20 cases in week four and 17 cases in week eight. In intention-to-treat analysis, all-cause mortality incidence rates were 25 per 100 person-years (95% CI 17.1–36.3) in week one, 18 per 100 person-years (95% CI 11.3–27.2) in week four and 15 per 100 person-years (95% CI 9.1–23.6) in week eight, P-value 0.2 by the log-rank test (Fig 2). All cause mortality incidence rate ratios in subgroups with baseline CD4 counts <50 cells/μL versus above or equals to 50 were 2.8 in week one (95% CI 1.2–6.7), 3.1 in week four (95% CI 1.2–8.6) and 5.1 in week eight (95% CI 1.8–16). Seventy-one cases (14.9%) were censored as Lost to follow up (LTF) which is defined as can’t be reached or outcome traced. The LTF rates were 26 cases (24 per 100 person-years) in week one, 24 cases (21 per 100 person-years) in week four and 21 cases (18 per 100 person-years) in week eight (P = 0.7 by the log-rank). In a sensitivity analysis assuming the LTF dead, all cause mortality incidence rates were 49 per 100 person-years (95% CI 37–64) in week one, 39 per 100 person-years (95% CI 29–52) in week four and 33 per 100 person-years (95% CI 24–45) in week eight, P = 0.2 by the log-rank test. Forty-three of the 64 deaths (67.2%) occurred within the first two months of TB therapy. At the end of the second month of TB therapy, all cause mortality incidence rates were 17.5 per 100 person-years in week one (19 cases, 95% CI 11.2–27.5), 12.3 per 100 patient years in week four (14 cases, 7.3–20.8) and 8.6 per 100 person-years (10 cases, 95% CI 4.6–16) in week eight, P-value 0.2 by the log-rank test. Twenty-two cases (34.4%) died before cART was initiated at their respective study weeks. The numbers of cases were 6/27 (22.2%) in week one, 7/20 (35%) in week four and 9/17 (52.9%) in week eight. Excluding the deaths before cART was started at each study week, all-cause mortality incidence rates were 19 per 100 person-years (95% CI 12.7–30) in week one, 11 per 100 person-years (95% CI 6.6–19.7) in week four and 7 per 100 person-years (95% CI 3.5–13.9) in week eight, P-value 0.03 by the log-rank test. Cox proportional-hazards regression analysis showed CD4 count < 50 cells/μL (adjusted HR, aHR = 2.7, 95% CI 1.6–4.5) and serum albumin less than 3gms/dL (aHR = 2.3, 95% CI 1.3–4) independently predicted mortality (Table 2).

**Table 2:** Risk factors for death among the 64 patients.

Risk factors	Univariate Analysis			Multivariate Analysis		
	Hazard Ratio (HR)	95% CI	P value	HR	95% CI	P value
**Baseline CD4 count**						
< 50 cells/ μL	3.3	2–5.4	0.001	2.7	1.6–4.5	0.001
51–199 cells/ μL	1			1		
**Serum Albumin**						
< 3gms/dL	2.8	1.6–4.9	0.001	2.3	1.3–4	0.02
≥ 3gms/dL	1			1		
**Randomization weeks**						
week one	1.6	0.9–3	0.1	1.5	0.8–2.7	0.2
week four	1.2	0.6–2.2	0.6	1.2	0.6–2.3	0.6
week eight	1			1		
**Body Mass Index (BMI)**	0.9	0.8–1	0.02	0.9	0.8–1	0.04
**Hepatotoxicity requiring interruption of TB therapy**	2	1.1–3.6	0.02	1.6	1–2.9	0.1

### Secondary endpoints

#### Drug-induced hepatotoxicity

During the study period 278 cases (58.2%) experienced one or more grade 1 and 2 hepatotoxicity events while 71 cases (14.9%) had grade 3 and 4 hepatotoxicity events (Table 3). The incidence rates of grade 1 and 2 hepatotoxicity events were 81 per 100 person-years (88 cases, 95% CI 66–100) in week one, 91 per 100 person-years (104 cases, 95% CI 75–110) in week four and 74 per 100 person-years (86 cases, 95% CI 60–91) in week eight, P value 0.1 by the log-rank test. The incidence rates of grade 3 and 4 hepatotoxicity events were 25 per 100 person-years (27 cases, 95% CI 17–36) in week one, 20 per 100 person-years (23 cases, 95% CI 13–30) in week four and 18 per 100 person-years (21 cases, 95% CI 12–28) in week eight, P- value 0.5 by the log-rank test (Table 4). Using the 2006 American Thoracic Society (ATS) criteria for hepatotoxicity, 49 cases (10.3%) interrupted first-line TB therapy. Twenty-two were in week one, 13 in week four and 14 in week eight. The median (range) duration of first-line TB treatment interruption was 14 days (4–30). The incidence rates of hepatotoxicity requiring first-line TB treatment interruption according to these criteria were not significantly different among study groups (P = 0.2, Table 4). However, in subgroup of patients with CD4 < 50cells/μL, the incidence rate of TB treatment interruption because of hepatotoxicity was highest in week one (P = 0.02, Table 4). Cox- multivariate regression analysis showed that positive HCV Ab test (aHR = 4.4, 95% CI 1.5–13) and CD4 count < 50 cells/μL (aHR = 2, 95% CI 1.2–3.2) were independent predictors of grade 3 or 4 hepatotoxicity when adjusted for the study randomization week, sociodemographic variables, BMI and serum albumin levels. From the 64 mortality cases, 16(25%) interrupted first-line TB therapy because of hepatotoxicity. Their respective numbers were 11(40.7%), 4(20%) and 1(5.9%) in weeks one, four and eight (P = 0.03).

**Table 3:** Drug-induced hepatotoxicity.

Random week	Hepatotoxicity		TB therapy interrupted due to hepatotoxicity. Total no, (%)	TB therapy interrupted due to hepatotoxicity (mortality cases, no(%)	Days TB therapy was interrupted due to hepatotoxicity, median (range)
	Grade 1 & 2	Grade 3 & 4			
Week one (n = 163)	88(54%)	27(16%)	22(12.3%)	11/27 (40.7%)	14(4–30)
Week four (n = 160)	104(65%)	23(14.4%)	13(8.1%)	4/20(20%)	15(9–30)
Week eight (n = 155)	86(55.5%)	21(13.5%)	14(9%)	1/17(5.9%)	14(8–30)
Total (n = 478)	278(58.2%)	71(14.9%)	49(10.3%)	16/64 (25%)	14(4–30)

**Table 4:** Summary of Efficacy and Safety endpoints.

Out comes	Frequency	week one				week four				week eight				P value
		no	per-yrs	no. events	event rate/100 per-yrs (95% CI)	no	per-yrs	no. events	event rate/100 per-yrs (95% CI)	no	per-yrs	no. events	event rate/100 per-yrs (95% CI)	
**All-cause mortality**	All patients	163	108.4	21	26(17.1–36.3)	160	114.5	20	18(11.3–27.2)	155	116.3	17	15(9.1–23.6)	0.2
	CD4 < 50	61	36.8	16	43.5(26.7–71)	50	32.4	11	34.5(19.1–62.2)	39	25.3	10	39.7(21.4–73.8)	0.9
	CD4 ≥ 50	102	36.8	11	15.4(8.5–27.7)	110	82.1	9	11(5.7–21.1)	116	91	7	7.7(3.7–16.2)	0.4
	IRR *(95% CI)				2.8(1.2–6.7)				3.1(1.2–8.6)				5.1(1.8–16)	
**Grade 3 & 4 hepatotoxicity** **	All patients	163	108.4	27	25(17–36)	160	114.5	23	20(13–30)	155	116.3	21	18(12–28)	0.1
	CD4 < 50	61	36.8	20	54.4(35.1–84.3)	50	32.4	5	15.4(6.4–37)	39	25.3	7	27.7(13.2–58.1)	0.01
	CD4 ≥ 50	102	36.8	7	9.8(4.7–20.5)	110	82.1	18	21.9(13.8–34.8)	116	91	14	15.4(9.1–26)	0.1
	IRR *(95% CI)				5.6(2.3–15.6)				0.7(0.2–2)				1.8(0.6–4.8)	
**TB therapy interrupted** ***	All patients	163	108.4	22	20.3(13.4–30.8)	160	114.5	13	11.4(6.6–19.5)	155	116.3	14	12(7.1–20.3)	0.2
	CD4 < 50	61	36.8	17	46.2(28.8–74.4)	50	32.4	4	12.3(4.6–32.9)	39	25.3	6	23.8(10.7–52.9)	0.02
	CD4 ≥ 50	102	36.8	5	7(2.9–16.8)	110	82.1	9	11(5.7–21.1)	116	91	8	8.8(4.4–17.6)	0.7
	IRR *(95% CI)				6.6(2.3–23)				1.1(0.3–4)				2.7(0.8–8.9)	
**TB-IRIS**	All patients	163	108.4	16	14.8(9–24)	160	114.5	6	5.3(2.3–11.7)	155	116.3	0		0.001
	CD4 < 50	61	36.8	16		50	32.4	6						
	CD4 ≥ 50	102	36.8	0		110	82.1	0						
**ADIs**	All patients	163	108.4	12	11.1(6.2–19.5)	160	114.5	14	12.2(7.2–20.6)	155	116.3	13	11.2(6.5–19.2)	0.9
	CD4 < 50	61	36.8	12		50	32.4	14		39	25.3	13		
	CD4 ≥ 50	102	36.8	0		110	82.1	0		116	91	0		
**Sputum converted negative#**	Total	no	Sample avail (no)	Negative sputum (no)		no	Sample avail (no)	Negative sputum (no)		no	Sample avail (no)	Negative sputum (no)		0.2
		29	18	18		28	23	22		42	37	36		
	CD4 < 50	10	6	6		10	6	5		14	11	11		
	CD4 ≥ 50	19	12	12		18	17	17		28	26	26		

* Incidence Rate Ratios between subgroups with CD4 counts below 50 and above or equals to 50 cells/μL

** According to the Division of AIDS Table for Grading of Severity of Adult and Pediatric Adverse events (DAIDS AE Grading Table), December 2004 revision,

*** First-line TB treatment interrupted according to American Thoracic Society (ATS) criteria, 2006

# Smear status for eight weeks after TB therapy in smear positive Pulmonary Tuberculosis cases

#### Tuberculosis Immune Reconstitution Inflammatory Syndrome (TB-IRIS)

Total of 22 cases (4.6%) were diagnosed with TB- IRIS. Sixteen (72.7%) had lymphadenopathy either newly emerging or pre-existing increased in size. Six (27.2%) had new or worsening abnormalities either on CXR or ultrasound. The incidence rates of TB-IRIS were 15 per 100 person-years in week one (95% CI, 9–24), 5 per 100 person-years in week four (95% CI, 2.3–11.7) and none in week eight (P-value 0.001 by the log-rank test). The baseline CD4 count was lower than 50cells/μL in the TB-IRIS cases. In none of the cases, the course of their treatment was altered. TB-IRIS was diagnosed only in one of the mortality cases. Seven of the cases (44%) with lymph node TB-IRIS required surgical drainage or aspiration of the abscess and non-steroidal anti-inflammatory drugs to alleviate their symptoms.

#### AIDS defining illnesses (ADIs)

There were 39 ADIs documented. These events occurred at median (range) of 4 weeks (1–20 weeks) after TB treatment was started. Twelve of the cases were in week one, 14 in week four and 13 in week- eight. The incidence rates were 11.1 cases per 100 person-years (95% CI 6.2–19.6) in week one, 12.2 cases per 100 person-years (95% CI 7.2–20.6) in week four and 11.2 cases per 100 person-years (95% CI 6.5–19.2) in week eight (P-value 0.9 by the log-rank test, Table 4). The diagnoses were sepsis (18 cases), PCP with or without superimposed pneumonia (12 cases), cryptococcal meningitis (6 cases) and CNS Toxoplasmosis (3 cases). Intervening new ADIs accounted for 39(61%) of the deaths.

#### HIV-related outcomes (CD4 counts increase and HIV-1 RNA response)

The median (IQR) CD4 count at 48 weeks was 237 cells/μL (155–342 cells/μL) and there was no significant difference among the different weeks in the median (IQR) CD4 counts increase at 48 weeks (P = 0.9). At 12 weeks, CD4 count increase was the lowest in week eight (P = 0.001) and at 24 weeks in week four (P = 0.001). Suppression of plasma HIV-1RNA levels to undetectable viral load (≤ to 400 copies/mL) was achieved in 91% of the 220 cases for whom data were available at 48 week. The hazard rate (HR) of not achieving undetectable viral load at 48 weeks was 6 per 100 person-years (95% CI 1.9–19) in week one, 12 per 100 person-years (95% CI 6–24) in week four and 12 per 100 person-years in week eight (95% CI 6–22), P-value 0.5 by the log-rank test. But at 24 weeks the HR were 7 per 100 person-years (95% CI 3–16) in week one, 23 per 100 person-years (95% CI 15–35) in week four and 27 per 100 person-years (95% CI 18–41) in week eight, P-value 0.004 by the log-rank test.

#### TB-treatment outcome (sputum smear conversion in SP-PTB)

Seventy-eight participants from the 99 SP-PTB cases (79%) were able to produce sputum at eight week of TB therapy and 76 (77%) had negative smear for AFB. The proportion of cases whose smear converted negative was not different among the study weeks (P = 0.2, Table 4). The two cases whose sputum failed to convert had confirmed multidrug resistant TB.

## Discussion

There are several new and important findings in our study related to initiation of cART one week after TB therapy compared with four and eight weeks. Our study results showed that initiation of cART one week after TB therapy was not associated with improved overall survival at 48 weeks in patients with CD4 count < 200cells/ μL. In contrary to our study hypothesis the mortality rate was higher in week one, albeit statistically not significant. Consistent with other studies [9,22], two-third of the mortality events in our study occurred within the first two months of TB therapy. One-third of the mortality events occurred before cART was initiated. Post-hoc analysis showed cases with absolute CD4 count less than 50cells/μL compared with more than or equals to 50cells/μL are five times as likely to die within 48 weeks when cART was deferred for eight weeks. Overall absolute CD4 counts less than 50 cells/μL and serum albumin level < 3gms/dL while initiating TB therapy were independent predictors of mortality in our study. Similar results were reported from other cohort studies [12,14,16,17]. The case fatality ratio (CFR) of 13.4% in our study was relatively higher than 5.8% reported by Abdool-Karim et al. in the SAPiT clinical trial[15] from South Africa and 9.2% reported by Straetemans et al. [23] from a pooled over data of eight studies in TB and HIV co infected patients. Most reports from the sub-Saharan African countries revealed CFR of 20% or less in coinfected patients not on antiretroviral therapy or deferred more than eight weeks [9]. As compared with studies that initiated cART within the first eight weeks of TB therapy [12,15,16], our reported mortality incidence rates that ranged from 15 to 25 per 100 person-years were higher. The relatively higher mortality rate in our study was not due to selection bias. It is explained by the low CD4 counts at baseline, the type of TB cases, incidence of new AIDS defining illnesses and other co morbidities, quality of medical care, socioeconomic as well as geographical differences. Half of our study patients had SN-PTB. The CFR for SN-PTB in HIV coinfected patients in some sub-Saharan African countries is two times more compared with SP-PTB coinfected cases [9]. It is partly because of misdiagnosis of other HIV-related diseases as TB. An earlier study from our main clinical trial site showed from 131 HIV-positive SN-PTB cases (median CD4 count of 82cells/μL), TB culture from bronchoalveolar lavage was positive only in 31(24%) of the cases [24].

When our study started, there were ongoing studies to evaluate cART timing in TB and HIV coinfection at different CD4 count thresholds. Blanc et al.[12]in the Cambodian Early versus Late introduction of Antiretrovirals (CAMELIA) study showed cART initiated within two weeks of TB therapy in confirmed TB cases as compared with eight weeks reduced all-cause mortality rate after a median follow up period of 25 months. The overall survival benefit in this study may be explained by median CD4 count of 25 cells /μL as compared with median CD4 count of 73 cells/μL in our study population. Abdool Karim et al. in SAPiT clinical trial [15] evaluated cART within 4 weeks compared with 8 to 12 weeks of TB therapy in confirmed HIV-associated TB cases. Havlir et al. [16] in ACTG A5221 clinical trial evaluated cART within 2 weeks compared with 8 to 12 weeks of TB therapy in confirmed and probable TB cases. Both studies did not show reduction of AIDS events or death, apart from patients with CD4 counts below 50cells/μL [15,16]. Survival benefit related with earlier cART was discernible only in subgroup with CD4 count less than 50cells/μL. Manosuthi et al. [17] in TIME study from Thailand demonstrated cART initiated (median CD4 count 43cells/μL) four weeks after TB therapy in confirmed TB cases as compared with 12 weeks was not associated with improved survival one year after enrollment. Han et al. [25] in TREAT Asia HIV study showed that the interval between cART initiation and TB therapy did not significantly impact all-cause mortality rate. Consistent with the above results, our study didn’t show association between cART initiated at a median of seven days following TB therapy and improved survival. The relatively higher mortality in week one is partly explained by increased proportion of cases with CD4 counts < 50cells/μL, which is independent risk factor for mortality as well as grade 3 and 4 hepatotoxicity.

Hepatotoxicity is the most frequent treatment limiting, overlapping toxicity in TB/HIV co- treatment [26]. In this study, we found no statistically significant difference in incidence rates of the various hepatotoxicity grades including first-line TB treatment interruption. In subgroup analysis of cases with CD4< 50 cells/μL, the incidence rates of grade 3 and 4 hepatotoxicity and TB-treatment interruption was significantly increased in week one. Eleven (41%) of the fatality cases in week one, significant number compared with weeks four and eight, interrupted first-line TB therapy due to hepatotoxicity. The TB treatment interruption and its prerequisite hepatotoxicity contributed to the increased mortality seen in week one. Torok et al. [27]in OXTREC 023–04 clinical trial (median CD4 count of 41 cells/μL) compared cART within seven days of TB treatment initiation with eight weeks after in TB meningitis cases. In this clinical trial, there wasn’t statistically significant difference in the proportion of cases with grade 3 and 4 hepatotoxicity between the two treatment arms. Consistent with this finding, between early and late treatment arms of the SAPiT, CAMELIA and ACTG A5221 clinical trials significant differences in grade 3 and 4 hepatotoxicity were not seen [12,15,16]. The evidence from these clinical trials indicates that treatment limiting hepatotoxicity is mainly related with first-line TB drugs rather than antiretroviral therapy or its timing. Similar conclusion was reported from another study [28]. Independent risk factors for grade 3 and 4 hepatotoxicity in our study are CD4 count less than 50cells/μL and positive HCV antibody test. Ungo et al. reported [29] the risk of developing TB drugs-induced hepatotoxicity is 14.4 fold higher if there is HIV and HCV coinfection. In contrast to other studies [30,31], positive HBsAg status was not associated with increased risk of grade 3 and 4 hepatotoxicity in our study. The possible explanations are selection of tenofovir in the antiretroviral regimen of all HBsAg positive cases and inadequate study power to evaluate this effect.

Similar to other studies, the most frequent clinical manifestation of TB-IRIS in our study was the emergence of new or worsening preexisting lymphadenopathy [12,32]. The incidence rate TB-IRIS was significantly higher in week one compared with weeks four and eight. It is lower than 37.9 cases per 100 person-years reported from CAMELIA study [32] and 20.1 cases per 100 person-years reported from SAPiT trial [15] in earlier cART groups. In our study, TB-IRIS events were not documented in the mortality cases other than one. Some of the hepatotoxic events can be attributed to TB-IRIS [33,34]. The distinction between TB drugs induced hepatotoxicity and a hepatic TB-IRIS event is difficult because of similarities in clinical as well as laboratory manifestations.

The incidence rates of new ADIs were not significantly different between the various study weeks. The common finding was that the cases aggregated at CD4 count < 50cells/μL in all the study groups and the events occurred at a median of four weeks after TB therapy. New ADIs were reported in 60% of the deaths in our study. The findings imply the association between new ADIs and mortality events.

There was no significant difference with respect to median CD4 count increase and proportion of participants with undetectable viral load at 48 weeks. However, at 24 weeks HIV-related outcome measures were most favorable in week one. Our study findings suggest different exposure period to TB treatment at cART initiation doesn’t affect virologic suppression rate and CD4 count gains at 48 weeks. It is consistent with results from other studies [35].

Our study has strengths and limitations worth mentioning. One of the strengths is initiation of cART as early as one week after TB therapy. To our knowledge, the ACTG A5221 was the only other study to evaluate cART as early as median of 10 days after TB therapy. Both the confirmed and probable TB cases in our study represent the cases seen in low-income and high TB/HIV prevalent settings. The study has adequately addressed an important treatment limiting toxicity in managing TB/HIV coinfection. To this effect, liver function test was routinely performed at baseline, just before cART, every two weeks the first eight weeks and then every four weeks until week 48. The study limitations are: its open label design which can result in biased responses in safety assessment and reporting, the proportion of confirmed TB cases either with smear microscopy or histopathology and choosing all-cause mortality rate as primary end point rather than cause specific mortality rate. The latter was more appropriate to evaluate cART outcome. In spite of the differences observed in the number of deaths among the study groups, we failed to show statistical significance. It is possible that our study was not adequately powered to detect these differences. Irrespective of this, cART one week after TB therapy did not prove beneficial.

In summary with respect to the question of cART timing within the first two months of TB therapy, our study from low-income and high TB/HIV prevalent setting showed cART one week after TB therapy does not improve all-cause mortality rate in coinfected patients with CD4 count < 200 cells/μL compared with four and eight weeks. Majority of the deaths in our study occurred during the first two months of TB therapy. Deaths the first two months of TB therapy in HIV coinfected patients are more likely TB-related than other HIV-associated morbidities [36,37,38].

Therefore, the patient’s overall condition including other comorbidities is probably more important than the time to initiate cART in predicting outcome. Different studies including our have clearly shown CD4 count less than 50 cells/μL independently predicts mortality in TB/HIV coinfection. In our study, the mortality trend increased in this subset of patients as cART deferred from week one to eight. On the other hand, this same group of patients had the highest incidence rate of grade 3 and 4 hepatotoxicity and subsequent interruption of first-line TB therapy in week one. The key question for CD4 subgroup < 50 cells/μL is striking the optimal balance between the potential survival benefit if cART is initiated one week after TB therapy as opposed to the increased morbidity and mortality due to hepatotoxicity and risk of TB treatment interruption. Based on our findings, we recommend cART later than the first but earlier than the fourth week of TB therapy in subset of patients with baseline CD4 count less than 50 cells/μL.

## Supporting Information

S1 CONSORT Checklist (PDF)

S1 Data The whole data of the three study groups excluding Hepatotoxicity. (XLSX)

S2 Data The three study groups Hepatotoxicity data. (XLSX)

S1 Protocol Original Study Protocol. (DOC)
