# Supplementary material for: Efficacy and Safety of Antiretroviral Therapy Initiated One Week after Tuberculosis Therapy in Patients with CD4 Counts < 200 Cells/μL: TB-HAART Study, a Randomized Clinical Trial
Source: PLoS One. 2015 May 12;10(5):e0122587. doi: 10.1371/journal.pone.0122587 (PMC4429073; doi:10.1371/journal.pone.0122587)
Supplement: S1 Protocol — (DOC) [file pone.0122587.s004.doc]

###### Randomized clinical trial to determine safety and efficacy of **immediate versus deferred initiation of HAART among treatment naïve HIV infected adults requiring treatment for smear positive pulmonary & extrapulmonary TB.**

###### Principal investigator: Wondwossen Amogne, MD

**Abiy Habtewold, Msc**

**A research proposal to be submitted to the graduate studies of Karolinska Institute as requirement for PhD thesis.**

###### Supervisors: Getachew Aderaye, MD

###### Eleni Aklilu, PhD

###### Lars Lindquist, MD, PhD

**Anders Sonnerborg, MD, PhD**

###### Alemayehu Worku, PhD

###### Table of contents Page Number

###### List of abbreviations 3

###### Summary 4

###### Back ground 5

###### Significance of the study 7

###### Study hypothesis 7

###### Specific study objectives 7

###### Study design 8

###### Study population 8

###### Sample size 9

###### Inclusion and Exclusion criteria 9

###### Clinical trial site 10

###### Study Period 10

###### Operational definition 11

###### Study protocol 12

###### Data analysis 13

###### Out come and end points 13

###### Limitation of the study 14

###### Project work plan 16

###### References 17

###### Questionnaire 19

###### Ethical consideration 27

###### Patient information sheet 31

###### Consent form 34

**List of Abbreviations**

AIDS Acquired Immuno Deficiency Syndrome

ADI AIDS defining illnesses

ATS American Thoracic Society

BHIVA British HIV Association

CDC United States Centres for Disease Control and Prevention

CD4 Cluster Determinant 4

CXR Chest X-ray

EFV Efavirenz

EMB Ethambutol

EPTB Extrapulmonary tuberculosis

HAART Highly Active Anti Retroviral Therapy

HIV Human Immunodeficiency Virus

IRIS Immune Reconstitution Inflammatory Syndrome

HAART Highly Active Antiretroviral Therapy

HR Fixed formulation tablets of Isoniazid and Rifampicin

NSAIDs Non Steroidal Anti-inflammatory Drugs

OIs Opportunistic Infections

PTB Pulmonary tuberculosis

RHZE Fixed formulation tablets of Rifampicin, Isoniazid, Pyrazinamide

and Ethambutol

RIF Rifampicin

SM Streptomycin

TB Tuberculosis

TB/HIV Tuberculosis and HIV co-infection

TB IRIS Tuberculosis Immune Reconstitution Inflammatory Syndrome

TST Tuberculin Skin Test

###### WHO World Health organization

###### Summary:

###### Background:

###### Tuberculosis (TB) is the most common opportunistic infection and cause of death in HIV infected patients. TB/HIV co-treatment is recommended in patients with low CD4 counts. The high death rate in the first 2 months of tuberculosis treatment provides an argument for antiretroviral therapy to be started as soon as possible. However, concurrent treatment of TB & HIV infection is challenging, given drug-drug interactions, overlapping toxicities, non adherence to treatment and worsening of clinical symptoms due to TB IRIS. Clear definition of the best time to initiate antiretroviral treatment in patients with tuberculosis awaits results from controlled trials.

###### Methods

###### This is a three arm interventional, randomized, open label, active control, parallel assignment study to evaluate the safety and efficacy of immediate as opposed to deferred HAART among TB/HIV co-infected patients with active TB and CD4 counts less than 200 cells/mm^3^. In each of the study arms 150 patients will be assigned and the duration of study will be for 48 weeks after initial enrolment.

###### **Results**

###### The primary endpoints are survival without AIDS progression and the occurrence of AIDS defining illnesses. The secondary end points will be incidence, morbidity and mortality associated with TB IRIS, frequency and severity of adverse drug reactions, efficacy of antiretroviral therapy as measured with viral load and CD4 counts as well as treatment adherence to anti TB and HAART. Study end points will be analyzed by intention to treat analysis.

###### **Ethical considerations:**

###### Patients who fulfil the inclusion criteria will be approached regarding participation. Should they not wish to take part in this study, this will not prejudice their clinical care. Written informed consent will be taken and patient confidentiality maintained. An independent data and safety monitoring board will be established.

###### **Conclusion:**

###### Results from the study will help to establish better TB/HIV treatment strategies and guidelines.

###### Background:

###### TB is the most frequent opportunistic infection in HIV infected patients and 70% of TB/HIV co-infected patients live in sub-Saharan Africa (1). Co-infection with TB is the leading cause of mortality among people living with HIV/AIDS, therefore, concomitant TB/HIV treatment is recommended in patients with low CD4 cell counts. Up to 70% of patients with sputum smear-positive pulmonary tuberculosis are HIV-positive in some countries in sub-Saharan Africa (2). Our previous study in Ethiopia indicted that 60% of TB diagnosed patients are HIV positive with CD4 cell count profile <50, 50-200 and >200/mm^3^ being 17, 62 and 21 % respectively, indicating that about 80% of these patients need concomitant HAART and anti-TB treatment (manuscript in preparation). For patients with active TB in whom HIV infection is diagnosed and HAART is required the first priority is to initiate standard antituberculosis treatment. Clear definition of the best time to initiate antiretroviral treatment in patients with tuberculosis awaits results from controlled trials (3). Case-fatality rates in patients with TB during the first two months of TB treatment are high, particularly in settings where there are high prevalences of HIV, suggesting that ART should begin early. On the other hand, considerations of pill burden, drug-drug interactions, toxicity and TB IRIS support the later initiation of HAART (3). There fore the time to initiate HAART in patients with TB/HIV co-infection is a balance between the risk of HIV progression if HAART is delayed against the risk of discontinuing therapies because of toxicities, side effects, TB IRIS or unforeseen drug-drug interactions. WHO guidelines suggest starting HAART within 2-8 weeks of anti TB treatment at a CD4 count of 200cells/mm^3^ or less and for extrapulmonary TB or other manifestations of severe immunosuppression (4). The CDC/ATS recommendation is to avoid initiating treatment of both and always to treat TB first with HAART introduced at 4-8 weeks later. The possible exception being patients with advanced HIV infection and CD4 count < 50 cells/ mm^3^ in whom HAART can be initiated in the first 2 weeks of antiTB therapy (5). The BHIVA guideline for TB/HIV co-infection recommends initiating HAART as soon as possible depending on the physician’s assessment following the start of anti TB therapy in patients with

###### CD4 count less than 100cells/mm^3^ (6). Few studies addressed the problem in patients with CD4 cell count <100 cells/mm^3^ supporting either immediate or deferred HAART.

###### Some experts recommend HAART to be started early in patients with advanced HIV diseases and deferring HAART until the continuation phase of TB therapy (i.e. after 2 months) for patients who are clinically stable with TB treatment (7).The evidences for the above arguments are not strong and the recommendations have been changing form time to time. The guidelines are largely based on expert’s opinion, data from small studies in developed countries, and a few pilot studies in developing countries (8, 9). Clinical data are lacking whether delaying initiation of HAART by 2- 8 weeks is advantageous or deleterious to the outcome of both the TB and the patient in general. It awaits results from controlled trials (5). The issues are complex, and a randomized trial is needed to clarify how the timing of HAART affects the competing risks of HIV disease progression versus those of drug side effects or a severe immune reconstitution events. In the present study, we will try to investigate the optimal time to initiate HAART in HIV-positive patients with active TB and CD4 count < 200 cells/mm^3^. The study has three arms in to which patients will be assigned randomly. In the first arm EFV based HAART will be initiated one week after anti TB medications are started and in the 2^nd^ and 3^rd^ arms at 4 weeks and 8 weeks following initiation of anti TB therapy. It will help to address some of the important challenges in the management of HIV-related tuberculosis particularly at resource limited settings. Globally, Ethiopia ranks the 4^th^ largest country affected by HIV/AIDS and the 8^th^ among the 22 high TB burden countries. In Addis Ababa region where the study is planned to be conducted, TB/HIV collaborative activity has been implemented as of February 2006 in 7 hospitals, 24 health centers, and 2 clinics. During the same period among 3564 TB patients who had been screened for HIV, 1439(40.3%) were found HIV positive (data from the regional health bureau). This clearly indicates need for optimization of TB & HIV co treatment. HAART was officially started in Ethiopia out of pocket funding basis in 2003 and free ART program was launched in January 2005. With rapid scale-up of antiretroviral therapy in countries like Ethiopia, management of TB/HIV coinfection will be a challenge.

###### **Significance of the study**

###### ****Antiretroviral therapy in individuals undergoing treatment for TB merits special consideration because co management of HIV and TB is complicated by: drug interactions between RIF and EFV, Immune Reconstitution Inflammatory Syndrome (IRIS); overlapping toxicities and adherence issues.****

###### ****Results of this study will have clinical relevance in understanding the optimal time to initiate HAART during anti TB therapy in patients with low CD4 counts.**** Identification of the optimal time to initiate HARRT during TB treatment will improve the treatment outcome of patients with TB/HIV co-infection. ****The final results of this study will help policy makers in Africa establish effective treatment guideline and strategies for TB/HIV co-treatment.****

###### **Study hypothesis**

###### ****The**** study hypothesis is immediate EFV based HAART initiated one week after anti TB treatment (arm-A) will reduce the frequency of other AIDS-defining illnesses and all cause mortality among TB/ HIV co-infected patients with base line CD4 counts < 200 cells/mm^3^ as compared to deferred HAART initiated either at 4 weeks of anti TB treatment (arm-B) or after 8 weeks as soon as the intensive phase of anti TB treatment is completed (arm-C).

###### Specific objectives:

###### To evaluate the effect of immediate as opposed to deferred initiation of HAART

###### in TB/HIV co infected Patients with active TB and CD4 count < 200 cells/mm^3^ on:

1. The incidence of new AIDS Defining illnesses (ADI)

2. All cause mortality

3. Efficacy of HAART as measured by viral load suppression.

4. Efficacy of HAART as measured by CD4 increase.

5. Incidence of TB IRIS

6. Potential morbidity associated with TB IRIS

7. Potential mortality associated with TB IRIS

8. Frequency and severity of adverse drug reactions related to anti TB medications.

9. Frequency and severity of adverse drug reactions related to antiretroviral drugs.

10. Treatment adherence challenges related to anti TB and antiretroviral drugs.

**Study Design:** Interventional, randomized, open label, Active control, parallel assignment, safety and efficacy study

###### Study population:

###### At study entry participants will be randomly assigned to one of the three arms.

###### arm A (Immediate Treatment Group): Receipt of antiretroviral therapy one week after starting anti-TB treatment ("immediate" group)

###### arm B (Deferred Treatment Group-1): Antiretroviral therapy will be initiated at the 4^th^ week of starting anti-TB treatment

###### arm C (Deferred Treatment Group-2): Antiretroviral therapy will be initiated at the 8^th^ week of starting anti-TB treatment as soon as intensive phase is completed.

###### Sample Size:

###### TB case-fatality rates in Africa are 16-35% in HIV positive individuals not receiving HAART and 4-9% in HIV negative patients (3). We assumed 25% case-fatality rate among TB/HIV co-infected patients who will start HAART after 2 weeks of anti-TB

###### therapy and early initiation (< 2 weeks) will reduce this frequency to 13%. The minimum sample size required detecting the12% reduction in the case-fatality rate with 80% power, 5% type I error and 20% non-response rate is 440. There fore in each of the three study arms 150 patients will be assigned randomly.

###### Inclusion criteria:

######

###### 1. Confirmed HIV infected patients of both sexes and age > 18years.

###### 2. Clinical and/or radiological pictures consistent with TB and two sputum samples

###### positive for AFB.

3. Sputum or any other body fluid culture result positive for Mycobacterium tuberculosis.

4. Tissue biopsy of FNAC results consistent with the diagnosis of tuberculosis

3. CD4 cell count < 200/mm^3^ at the time of TB diagnosis

4. Residence in Addis Ababa

5. Ability to give signed written / thumb sign informed consent.

###### Exclusion criteria:

###### 1. Pregnancy and breast-feeding women

###### 2. Patients previously treated with anti TB drugs.

###### 3. Patients with previous treatment experience for HAART.

###### 4. TB/HIV co-infected patients receiving other medications that are contraindicated

###### or not recommended to be used with EFV or RIF.

###### 5. Severely ill patients Karnofsky performance status score < 40.

6. Patients with known active or chronic active liver disease in whom avoidance of

INH & Rifampicin is indicated.

###### 7. Anyone who does not meet the inclusion criteria

**Clinical Trial sites:** Tikur Anbessa Specialized Referral Hospital which is the main teaching and referral hospital for Addis Ababa university, Medical Faculty and St. Peter’s TB specialized Hospital will be the main study sites. Patients will also be recruited from public health facilities in the Addis Ababa region.

**Study period:** The total study duration is expected to be 2 years (1 year for enrolment, one year of follow-up, and lab work) in the two study sites to run in parallel.

###### Operational definition:

###### 1. Pulmonary Tuberculosis (PTB) = Clinical illness consistent with active TB and

###### at least two initial smear examinations positive for AFB by direct microscopy

###### or culture-positive.

###### 2. Extrapulmonary Tuberculosis (EPTB) = TB in organs other than the lungs

###### proven by one culture positive specimen from an extra-pulmonary site or

###### histopathological evidence from a biopsy or Fine Needle Aspiration Cytology.

###### 3. Tuberculosis Immune Reconstitution Inflammatory Syndrome (TB IRIS) =

###### There is no validated clinical case definition of TB IRIS. The following criteria

###### will be used to diagnose TB IRIS in our study following initiation of EFV

###### based HAART at confirmed TB/HIV cases who have already started taking

###### anti-TB treatment (10, 11, 16). The diagnosis of TB IRIS would require at

###### least one major criterion and two minor criteria including fall in the viral load

###### by one or more log.

**Major criterion**

**Any one of the following:**

- New persistent fever without an identifiable source or reason.
- New or worsening enlarging lymph node/s or cold abscess.
- New focal tissue or organ involvement with TB.
- New or worsening radiological features of TB.
- Breakthrough TB meningitis or new focal CNS lesion.
- New or worsening serositis ( pleural, ascites, pericardial, arthritis)

Plus

- > 1 log decrease in plasma viral load.

**Minor criterion**

- Increase in CD4 cell count of 25 cells/mm^3^
- Biopsy demonstrating well-formed granulomatous inflammation or unusually exuberant inflammatory response
- Worsening or emergence of cough, dyspnea and/or stridor
- New or worsening abdominal pain with ultrasound evidence of abdominal lymphadenopathy or hepatosplenomegally
- Resolution of clinical + radiological findings without additional/ change in anti TB therapy
- Positive TST

**Exclude the following conditions.**

- Anti TB failure secondary to drug resistance
- New OI or neoplasm
- Adverse drug reaction
- In adequate adherence to HAART

###### 4. Tuberculosis is classified as localized or as disseminated if it involves

###### > 2 organs.

###### Study protocol:

###### After explaining the purpose of the study at an individual level to confirmed new cases of PTB and EPTB, pre-test counselling will be offered to those who consent screening with rapid HIV tests. The diagnosis of HIV will be made based on the national HIV diagnosis algorithm. First blood will be tested with Determine® kit and if it is negative, the result will be interpreted as negative. If the result turns positive, it will be reconfirmed with Cappilus® kit and if this is positive, the patient will be labelled as positive. If the result turns out to be indeterminate, Unigold® kit will be used as tie-breaker. Following post-test counselling with prior informed written or thumb signed consent those who fulfil the study inclusion criteria will be enrolled in the study. Before anti TB or HAART is started, all the required baseline laboratory investigations will be determined. Patients will be randomly assigned to one of the three study arms as indicated in the study population. The random assignment will be done by lottery method. The intensive phase of anti TB therapy consists of 2 months treatment with Rifampicin, Isoniazid, Pyrazinamide and Ethambutol (RHZE). The continuation phase will be with Rifampicin and Isoniazid (RH) daily for 4 months. Directly Observed Therapy- Short Course (DOTS) will be the strategy that we shall follow strictly during the intensive phase of anti TB therapy. During the continuation phase of anti TB therapy the DOTS strategy will be modified with providing the drugs every week. Treatment adherence will be monitored by pill counting every week visit. The study will provide fixed formulations of RH and supplemental Vitamin B6 -50 mgs daily. RH daily given under direct observation for four months is the preferred continuation phase (12). Short course regimens that contain RIF through out give a better response and survival in HIV infected individuals and might reduce the risk of relapse (13, 14). EFV based HAART will be initiated with Zidovudine or Stavudine plus Lamivudine as the nucleoside back bone. The timing to initiate HAART will be according to the specific arm the patient will be randomly assigned (see flow chart below). Before HAART is initiated both CBC and blood chemistry results will be re-evaluated. Repeat CD4 and Viral load will be performed for those who will initiate EFV based HAART at study weeks 4 and 8. Primary prophylaxis will be offered with Cotrimoxazole 960 mgs tab daily for all patients with no known drug hypersensitivity at the same time with the start of anti TB medications.

###### Before HAART is initiated if a study subject develops Drug-induced hepatitis (DIH) defined as serum AST/ALT level more than three times the upper limit of normal in the presence of symptoms, or more than five times the upper limit of normal in the absence of symptoms then all the anti TB drugs will be discontinued & initiation of HAART will be deferred. LFT values will be monitored closely every one week and arrangements will be made for patients who require in hospital care to be admitted. Once the AST level decreases to less than two times the upper limit of normal and symptoms have significantly improved the anti TB drugs will be started in sequential manner. First INH 50 mgs will be started which will be increased to 100 mgs daily after 3 days then followed sequentially by RH, RHZ and RHZE full dose at an interval of 5-7 days. Seriously ill patients who require continuation of anti TB medications without hepatotoxicity such as EMB, SM or fluoroquniolone without RIF & INH will be excluded from the study. If a study subject either in arm A or B becomes unable to continue anti TB treatment because of side effects and/or develops grade 2 hepatitis without symptoms, HAART will be deferred and patient will be transferred to the next arm for safety reasons. During analysis these patients will be considered as failure of that specific arm. CD4 and viral load will be determined before study enrolment and then every three months until the 48^th^ to 52^nd^ weeks. As TB IRIS is suspected, both CD4 and viral load will be determined. Patients who will develop moderate to severe TB IRIS and whose symptoms can not be controlled with NSAIDs, Prednisolone 0.5mg/Kg/day for 5 to 10 days will be used(4 ). Indications to temporarily discontinue or substitute HAART will be Steven-Johnson’s syndrome, grade 4 hepatotoxicity, presumed lactic acidosis, peripheral neuropathy with intolerable symptoms, anaemia(Hgb< 8gms%) and pancytopenia. Reinstituting anti TB and switching the antiretroviral medications will be at discretion of the treating physicians.

TB/HIV co-infected patients with CD4 < 200 cells/mm3

(Baseline lab: CBC, Chemistry, HBsAg, HCV Ab, VL, TST)

Initiate Anti TB (Cat I) = 2RHZE + 4 HR

+ TMP-SMX prophylaxis

Randomize patients in to the trial arms

↓

arm A arm B arm C

EFV based HAART EFV based HAART EFV based HAART

One week after anti TB 4 weeks after anti TB 8 weeks after anti TB

↓ ↓

Unable to tolerate anti TB Unable anti TB

LFT > Grade 2 LFT > Grade 2

↓ ↓

Decide continuing anti TB Decide anti TB

Weekly LFT Weekly LFT

Manage DIH Manage DIH

Transfer arm B Transfer arm C

###### Data analysis

###### Data will be collected using prepared questionnaire and formats. It will be double entered and analyzed with SPSS version 12.0 statistical software. Categorical variables will be compared using X^2^ or Fisher’s exact test whereas non-categorical variables will be compared using Wilcoxon’s rank-sum test. Survival probabilities will be estimated by the method of Kaplan-Meier. Multivariate regression analysis will be done. Outcome variables in each of the study arms will be analyzed by intent to treat analysis. A p-value less than 0.05 will be considered as significant.

###### Outcomes and end points

###### With respect to the following outcome variables at the 8^th^, 24^th^ and 48 weeks, the percent of subjects in the immediate HAART therapy arm versus deferred HAART therapy arms will be compared. The Primary end points will be

###### 1. All cause mortality

###### 2. Incidence of new AIDS defining illness/es.

3. Morbidity due to new AIDS defining illness/es

4. Mortality due to new AIDS defining illness/es

The secondary end points will be safety and efficacy of treatment. Proportion of patients with virologic success defined as undetectable HIV RNA as measured by plasma HIV 1-RNA level, virologic failure (defined by two consecutive measurements > 50 copies/ml from week 24 on wards; a single plasma HIV-1 RNA concentration > 50 copies per ml at week 48), time to reach full suppression of the viral load, mean increase in the CD4 count and adverse events such as peripheral neuropathy, CNS adverse effects and hepatotoxicity will be recorded. The incidence of TB IRIS as well as morbidity and mortality related to it will be analyzed. Rate of sputum smear conversion will be compared among the different arms at second & fifth months following anti TB therapy. The level of treatment adherence both to anti TB and HAART versus pill burden will also be analyzed.

###### Limitation of this study:

###### There is no standard or universally accepted case definition of TB IRIS. The problems in developing clinically useful case definition include the following. It is mainly a clinical diagnosis with a wide variety of underlying OIs associated with it. The need to incorporate both unmasking of clinically silent TB and worsening of previously diagnosed

###### TB, and the difficulty to exclude that a new microbial process or drug toxicity is not the cause of a particular patient’s clinical presentation are the main challenges. Excluding drug toxicity or a new OI is always a diagnostic difficulty. It is even worse in countries with limited resources. The diagnosis of TB IRIS must be one of exclusion as it can be confused with recrudescence of TB due to treatment failure and drug hypersensitivity reaction. How ever the difficulty is that the clinical manifestations are non- specific and some are not easy to diagnose. To minimize the effects of the above issues in our study, we set an operational definition for TB IRIS. As TB/HIV co-infection often occurs on a background of advanced immunosuppression at CD4 counts < 200 cells/mm^3^, other opportunistic infections can occur during the follow up period the treatment of which can increase drug-drug interactions, drug toxicities and hence can potentially confound the study outcome. Some of the important toxicities related to TB and HIV treatment are overlapping; there fore it can be difficult to establish direct cause and effect relationship between a drug and subsequent toxicity. DOTS is the national strategy we are going to follow strictly with anti TB therapy during the intensive phase & it will be modified during the continuation phase with providing the drugs every week while assessing treatment adherence by pill counting. Where as the antiretroviral drugs will be provided every month for the patients with adequate instructions to take it at home. We will try to assess treatment adherence to the anti TB drugs during the continuation phase and the antiretroviral drugs by counting the pills and based on patients self reporting. This method of monitoring treatment adherence has its own limitations for not being the best parameter to assess treatment adherence reliably.

Project work plan

| **Phase** | **Month** | **Description** |
| --- | --- | --- |
| **1** | **November 2007** | **Project fund approval,** |
| **2** | **January 2008** | **Proposal submission for National Ethical clearance** |
| **3** | **March 2008** | **Ethical clearance** |
| **4** | **April 2008-**  **May 2008** | **Research staff recruitment, trial site infrastructure up grading, Course in GCP, GLP, SOP & ethics etc** |
| **5** | **June 2008-**  **Dec 2009** | **Patient identification, follow up, medical history taking, physical examination, blood sample collection, laboratory studies including HPLC, plasma HIV RNA quantification, genotyping for CYP etc,** |
| **6** | **Jan 2010- May 2010** | **Finalizing patient follow up, lab work, Data entries, analysis and write up** |

References:

1 Dye C, Scheele S, Dolin P, Pathania V, Raviglione MC. Consensus statement. Global burden of tuberculosis: estimated incidence, prevalence, and mortality by country. WHO Global Surveillance and Monitoring Project.

*JAMA* 1999; 282:677-686.

2 Raviglione MC, Harries AD, Msiska R, Wilkinson D, Nunn P. Tuberculosis and HIV: current status in Africa. *AIDS* 1997; 11 Suppl B:S115-123.

3. Corbett EL, Marston B, Churchyard GJ, De Cock KM. Tuberculosis in sub-Saharan Africa: opportunities, challenges, and change in the era of antiretroviral treatment. *Lancet* 2006; 367:926-937.

4. World Health Organization, Antiretroviral therapy for HIV infection in adults and adolescents in resource limited settings: Towards universal access, Recommendation for a public health approach, 2006 revision

5. Centers for Disease Control and Prevention. Treating opportunistic infections among HIV-infected adults and adolescents: recommendations from CDC, the National Institutes of Health, and the HIV Medicine Association/ Infectious Diseases Society of America. MMWR 2004; 53(No. RR-15). Atlanta, GA: CDC, 2004, 2004.

6. Pozniak AL, Miller RF, Lipman MC, Freedman AR, Ormerod LP, Johnson MA*, et al.* BHIVA treatment guidelines for tuberculosis (TB)/HIV infection 2005. *HIV Med* 2005; 6 Suppl 2:62-83.

7. Dean GL, Edwards SG, Ives NJ, Matthews G, Fox EF, Navaratne L*,*

*et al.* Treatment of tuberculosis in HIV-infected persons in the era of highly active antiretroviral therapy. *Aids* 2002; 16:75-83.

8 Blumberg HM, Burman WJ, Chaisson RE, Daley CL, Etkind SC, Friedman LN*, et al.* American Thoracic Society/Centers for Disease Control and Prevention/Infectious Diseases Society of America: treatment of tuberculosis.

*Am J Respir Crit Care Med* 2003; 167:603-662.

9. Jack C, Lalloo U, Karim QA, Karim SA, El-Sadr W, Cassol S*, et al.* A pilot study

of once-daily antiretroviral therapy integrated with tuberculosis directly observed

therapy in a resource-limited setting.

*J Acquir Immune Defic Syndr* 2004; 36:929-934.

10. Samuel A. Shelburne, Martin Montes, Richard J. Hamill. Immune Reconstitution Inflammatory Syndrome: more answers, more questions. *J Antimicrob Chemother2006; 57: 167-170*

11. R Colebunders, L. John, V Huyst, etal. Tuberculosis immune reconstitution inflammatory syndrome in countries with limited resources. Int J Tuberc Lung Dis 2006; 10: 946- 953

12 WHO/CDS/TB/2003. 313 Treatment of tuberculosis: guidelines for national programs, third edition, Revision approved by STAG, June 2004

13. Jindani A, Nunn AJ, Enarson DA. Two 8-month regimens of chemotherapy for treatment of newly diagnosed pulmonary tuberculosis: international multicenter randomized trial. Lancet 2004; 364:1244-51

14. Okwera A, Johnson JL, Luzze H et al. Comparison of intermittent

Ethambutol with Rifampicin- based regimens in HIV- infected adults with

PTB, Kampala. Int J Tuberc Lung Dis 2006; 10: 39-44

15. French M A, Price P, Stone S F. Immune restoration disease after

antiretroviral therapy. AIDS 2004; 18: 1615-1627

###### Randomized clinical trial to determine safety and efficacy of **immediate versus deferred initiation of HAART among treatment naïve HIV infected adults requiring treatment for smear positive pulmonary &**

###### **Extrapulmonary TB.**

**Confidential Case Record Forms**

**CRF - I. Identification:**

1. Date _____________ Visit No_________

2. Full Name____________________________

3. Address: Sub city: ________________ Kebele ________ House No _______

Telephone No________________

**CRF - II Demography:**

4.Date of birth: ______________

5. Sex F M

6. Marital status: Single Married Widowed Divorced

7. Education: Illiterate Able to read & write Primary School

Secondary School Tertiary

**CRF - III: Current symptoms check list**

| **Symptom** | **No** | **Yes** | **Duration/ Remark** | **Symptom** | **No** | **Yes** | **Duration/Remark** |
| --- | --- | --- | --- | --- | --- | --- | --- |
| Fever |  |  |  | Haemoptysis |  |  |  |
| Night sweats |  |  |  | Dyspnoea |  |  |  |
| Any weight loss (Kgs) |  |  |  | Headache |  |  |  |
| Nausea & Vomiting |  |  |  | Paraesthesia |  |  |  |
| Loss of appetite |  |  |  | Insomnia |  |  |  |
| Diarrhea (> 1 mo) |  |  |  | Hypersomnia |  |  |  |
| Cough ( > 2 wks) |  |  |  | Feels depressed? |  |  |  |
| Dyspahgia/odinophagia |  |  |  | Delusions |  |  |  |
| Facial swelling |  |  |  | Hallucinations |  |  |  |
| Skin rash |  |  |  | Suicidal ideation |  |  |  |

**CRF - IV Past medical history**

| **Past medical problem** | **No** | **Yes** | **Not recall** | **Specify status/ Organ/s involved** | **When?** |
| --- | --- | --- | --- | --- | --- |
| **PTB*** |  |  |  | AFB + ve AFB –ve Unknown |  |
| **EPTB**** |  |  |  |  |  |
| **ADI***** |  |  |  |  |  |
| **Hx of jaundice** |  |  |  |  |  |
| **Known CLD****** |  |  |  |  |  |
| **Blood transfusion** |  |  |  |  |  |
| **IPT ******* |  |  |  |  |  |
| **Hx of drug allergy** |  |  |  |  |  |
| **Previous Psychiatric problems requiring hospital visits/admission** |  |  |  |  |  |
| **Convulsion** |  |  |  |  |  |
| **Ever received ART for**  **PMTCT/PEP purposes** |  |  |  |  |  |
| **Smoking** | Current smoker Ex-smoker Never smoked    Number of Cigarettes/day= Time smoked= | | | | |
| **Significant alcohol intake©** |  |  |  | |  |

**Key: * PTB= Pulmonary TB, ** EPTB = Extra Pulmonary TB,**

***** ADI= Aids Defining Illness, **** CLD= Chronic Liver Disease,**

******* IPT= INH Preventive Therapy**

**© Significant alcohol intake is defined as follows. Hard liquor > 2 days/week or > 3 bottles of beer> 3 days/week or local alcohol (e.g. Tej, Katikala)> 2days/week the past one month.**

Other Past Medical Problems:

1._____________________________ 4. ___________________________

2. _____________________________ 5.____________________________

3. _____________________________ 6. ____________________________

**CRF - V: Obstetrics history (Females only)**

8. Pregnancy No Yes

**CRF -VI Present Diagnosis**

9. Type of TB Smear +ve PTB EPTB Disseminated TB

**(Please note that TB Pleurisy is reported as EPTB while Pleuroparenchymal PTB is reported as PTB.)**

10. How was the diagnosis of EPTB made?

FNAC Incisional biopsy Excisional biopsy Pleural biopsy

**CRF -VII Drug History**

11. Any drug/herb currently being taken by the patient No Yes

Specify 1.____________________________________________________________

2.____________________________________________________________

3.____________________________________________________________

**CRF -VIII Physical Examination**

**BP= __________mmHg Pulse = ____________ RR= ________**

**Temperature= ____________ Weight = _________ Height= _______**

**BMI = _____________ (will be calculated later by the study investigators)**

12. Conjunctival pallor No Yes

13. Icteric Sclera No Yes Unable to exclude

14. Oral candidiasis No Yes

15. Oral hairy leukoplakia No Yes

16. Significant lymphadenopathy No Yes, Localized Yes, Generalized

Specify the L/node areas _______________________________________________

17. Parotid enlargement No Yes, Unilateral Yes, bilateral

18. Chest Normal Crepitations Wheezing

Consolidation Pleural effusion

Conclusion: ______________________________________________________

_______________________________________________________

19. CVS Normal Valvular Heart Disease Cardiomegally

Pericardial rub Pericardial effusion

S3/ summation gallop ↑ JVP

Conclusion: ________________________________________________________

_______________________________________________________

20. Abdomen Normal Hepatomegally Splenomegally

Ascites Any palpable mass

Conclusion: ______________________________________________________

______________________________________________________

21. Integumentary system

Angular stomatitis No Yes

Seborrheic dermatitis No Yes

Herpes Zoster lesions/scar No Yes

(Specify the dermatome/s)

Chronic Herpes simplex lesions No Yes

**(Orolabial, genital or anorectal for > 1 mo duration)**

Papular pruritic eruption No Yes

Edema No Yes (specify)

Kaposis sarcoma No Yes (Stage)

(If yes, stage according to the ACTG criteria annexed below.)

**21. Neuropsychiatry evaluation**

Abnormal general appearance & behavior No Yes (specify)

Level of Alertness Alert Sleepy Unresponsive (specify)

Orientation to Time & Place No (specify) Yes

(Day, Date, Month, Year, Name of the health institution)

Speech abnormality No Yes (specify)

Mood abnormality No Yes (specify)

(Depressed/Elated)

Thought abnormality No Yes (specify)

Does the patient have insight? No Yes

Able to count from 20 back wards No Yes

to 1 or the days of the week backwards

Cranial nerve lesion/s No Yes (specify)

Lost of muscle power No Yes (specify)

Abnormal DTR No Yes (specify)

Sensory loss No Yes (specify)

Coordination abnormality/ies No Yes (specify)

Movement disorder No Yes (specify)

Meningeal signs No Yes (specify)

Conclusion: ________________________________________________________

________________________________________________________

_________________________________________________________

_________________________________________________________

**CRF -IX Diagnosis**

23. Type of TB =_____________________________________________

24. List other diagnosis.

1. ______________________________________________________

2. ______________________________________________________

3. ______________________________________________________

4. ______________________________________________________

25. WHO clinical stage (1-4)

26. Date of HIV diagnosis _______________

dd mm yyyy

27. Karnofsky score ____________________

28. CD4 count before HAART initiation ______________

29. HIV RNA before HAART initiation ______________

**CRF -X Treatment**

30. When was the intensive phase of anti TB started with “RHZE”? _____/ _____/ ______

dd mm yyyy

31. Co-Trimoxazole 960 mgs daily initiated as prophylaxis? 1/ Yes 2/ No

32. When was Co-trimoxazole initiated? ______/ ______/ _______

dd mm yyyy

33. When was patient started on HAART?

1. After one week of anti TB _______/ ______/_________

dd mm yyyy

2. After four weeks of anti TB ______/ ______/ _________

dd mm yyyy

3. After eight weeks of anti TB _____/ ______/ _________

dd mm yyyy

34. HAART regimen initiated 1/ d4T_30_/3TC/EFV 2/ d4T_40_/3TC/EFV 3/ CBV/EFV

35. List medications currently taken including the dose other than the anti retroviral drugs.

| Drug | Dose | Drug | Dose |
| --- | --- | --- | --- |
| 1. |  | 4. |  |
| 2. |  | 5. |  |
| 3. |  | 6. |  |

| **Laboratory Follow up - HAART at 1^st^ week** | | | | | | | | | | | | | | | | |
| --- | --- | --- | --- | --- | --- | --- | --- | --- | --- | --- | --- | --- | --- | --- | --- | --- |
| **Type of Lab** | **Normal range** | **Base line** | **1^st^**  **week** | **2^nd^ week** | **3^rd^ week** | **4^th^**  **week** | **6^th^**  **week** | **8^th^**  **week** | **12^th^ week** | **16^th^**  **week** | **20^th^**  **week** | **24^th^**  **week** | **28^th^**  **week** | **36^th^**  **week** | **48^th^ week** | **52**  **week** |
| **Hgb** | **12.5-17 g/dL** |  |  |  |  |  |  |  |  |  |  |  |  |  |  |  |
| **WBC** | **4.0-10.5x 10^3^** |  |  |  |  |  |  |  |  |  |  |  |  |  |  |  |
| **Neutrophils** | **1.8-7.8 x 10^3^** |  |  |  |  |  |  |  |  |  |  |  |  |  |  |  |
| **ESR** | **0-20 mm1^st^ hr** |  |  |  |  |  |  |  |  |  |  |  |  |  |  |  |
| **Urine HCG** | **Negative** |  |  |  |  |  |  |  |  |  |  |  |  |  |  |  |
| **Platelet** | **140-400x 10^3^** |  |  |  |  |  |  |  |  |  |  |  |  |  |  |  |
| **Sputum AFB** | **Negative** |  |  |  |  |  |  |  |  |  |  |  |  |  |  |  |
| **FBS** | **110mgs/dL** |  |  |  |  |  |  |  |  |  |  |  |  |  |  |  |
| **AST** | **0-60 iu/L** |  |  |  |  |  |  |  |  |  |  |  |  |  |  |  |
| **ALT** | **0-50 iu/L** |  |  |  |  |  |  |  |  |  |  |  |  |  |  |  |
| **ALP** | **0-483 iu/L** |  |  |  |  |  |  |  |  |  |  |  |  |  |  |  |
| **Bil (T)** | **0.1-1.1mg/dL** |  |  |  |  |  |  |  |  |  |  |  |  |  |  |  |
| **Bil (D)** | **0-0.4 mg/dL** |  |  |  |  |  |  |  |  |  |  |  |  |  |  |  |
| **T.Protein** | **6-8 g/dL** |  |  |  |  |  |  |  |  |  |  |  |  |  |  |  |
| **Albumin** | **3.5-5.5 g/dL** |  |  |  |  |  |  |  |  |  |  |  |  |  |  |  |
| **Urea** | **5-18 mg/dL** |  |  |  |  |  |  |  |  |  |  |  |  |  |  |  |
| **Creatinine** | **0.61.1mg/dL** |  |  |  |  |  |  |  |  |  |  |  |  |  |  |  |
| **HBsAg** | **Negative** |  |  |  |  |  |  |  |  |  |  |  |  |  |  |  |
| **HCV Ab** | **Negative** |  |  |  |  |  |  |  |  |  |  |  |  |  |  |  |
| **VDRL** | **Nonreactive** |  |  |  |  |  |  |  |  |  |  |  |  |  |  |  |
| **CD4** | **500-1400** |  |  |  |  |  |  |  |  |  |  |  |  |  |  |  |
| **HIV RNA** | **LDL** |  |  |  |  |  |  |  |  |  |  |  |  |  |  |  |
| **CXR (PA)** | **Normal** |  | **Base line CXR (PA) is required for all cases enrolled in the study. It is repeated only when required.** | | | | | | | | | | | | | |
| **TST** |  |  | **TST will be repeated only when IRIS is suspected or diagnosed.** | | | | | | | | | | | | | |
| **Abd. Sono*** | **Normal** |  | **Abdominal sonography will be done only if it is required.** | | | | | | | | | | | | | |
| **Note: Shaded zones the specific lab tests will not be done unless indicated. RFT will be repeated only if required.** | | | | | | | | | | | | | | | | |

**HAART at the 1^st^ week**

| **Laboratory Follow up -HAART at 4^th^ week** | | | | | | | | | | | | | | | | |
| --- | --- | --- | --- | --- | --- | --- | --- | --- | --- | --- | --- | --- | --- | --- | --- | --- |
| **Type of Lab** | **Normal**  **range** | **Base line** | **1^st^**  **week** | **2^nd^**  **week** | **3^rd^**  **week** | **4^th^**  **week** | **6^th^**  **week** | **8^th^**  **week** | **12^th^**  **week** | **16^th^ week** | **20^th^**  **week** | **24^th^**  **week** | **28^th^**  **week** | **36^th^**  **week** | **48^th^**  **week** | **52^nd^**  **week** |
| **Hgb** | **12.5-17 g/ dL** |  |  |  |  |  |  |  |  |  |  |  |  |  |  |  |
| **WBC** | **4.0-10.5x 10^3^** |  |  |  |  |  |  |  |  |  |  |  |  |  |  |  |
| **Neutrophils** | **1.8-7.8 x 10^3^** |  |  |  |  |  |  |  |  |  |  |  |  |  |  |  |
| **ESR** | **0-20 mm1^st^ hr** |  |  |  |  |  |  |  |  |  |  |  |  |  |  |  |
| **Urine HCG** | **Negative** |  |  |  |  |  |  |  |  |  |  |  |  |  |  |  |
| **Platelet** | **140-400x 10^3^** |  |  |  |  |  |  |  |  |  |  |  |  |  |  |  |
| **Sputum AFB** | **Negative** |  |  |  |  |  |  |  |  |  |  |  |  |  |  |  |
| **FBS** | **110mgs/dL** |  |  |  |  |  |  |  |  |  |  |  |  |  |  |  |
| **AST** | **0-60 iu/L** |  |  |  |  |  |  |  |  |  |  |  |  |  |  |  |
| **ALT** | **0-50 iu/L** |  |  |  |  |  |  |  |  |  |  |  |  |  |  |  |
| **ALP** | **0-483 iu/L** |  |  |  |  |  |  |  |  |  |  |  |  |  |  |  |
| **Bil (T)** | **0.1-1.2mg/dL** |  |  |  |  |  |  |  |  |  |  |  |  |  |  |  |
| **Bil (D)** | **0-0.4 mg/dL** |  |  |  |  |  |  |  |  |  |  |  |  |  |  |  |
| **T.Protein** | **6-8 g/dL** |  |  |  |  |  |  |  |  |  |  |  |  |  |  |  |
| **Albumin** | **3.5-5.5 g/dL** |  |  |  |  |  |  |  |  |  |  |  |  |  |  |  |
| **Urea** | **5-18 mg/dL** |  |  |  |  |  |  |  |  |  |  |  |  |  |  |  |
| **Creatinine** | **0.6-1.1mg/dL** |  |  |  |  |  |  |  |  |  |  |  |  |  |  |  |
| **HBsAg** | **Negative** |  |  |  |  |  |  |  |  |  |  |  |  |  |  |  |
| **HCV Ab** | **Negative** |  |  |  |  |  |  |  |  |  |  |  |  |  |  |  |
| **VDRL** | **Non reactive** |  |  |  |  |  |  |  |  |  |  |  |  |  |  |  |
| **CD4** | **500-1400** |  |  |  |  |  |  |  |  |  |  |  |  |  |  |  |
| **HIV RNA** | **LDL** |  |  |  |  |  |  |  |  |  |  |  |  |  |  |  |
| **CXR (PA)** | **Normal** |  | **Base line CXR (PA) is required for all cases enrolled in the study. It is repeated only when required.** | | | | | | | | | | | | | |
| **TST** |  |  | **TST will be repeated only when IRIS is suspected or diagnosed.** | | | | | | | | | | | | | |
| **Abd. Sono*** | **Normal** |  | **Abdominal sonography will be done only if it is required.** | | | | | | | | | | | | | |
| **Note: Shaded zones the specific lab tests will not be done unless indicated. RFT will be repeated only if required.** | | | | | | | | | | | | | | | | |

**HAART at the 4^th^ week**

| **Laboratory Follow up -HAART at 8^th^ week** | | | | | | | | | | | | | | | | |
| --- | --- | --- | --- | --- | --- | --- | --- | --- | --- | --- | --- | --- | --- | --- | --- | --- |
| **Type of Lab** | **Normal range** | **Base line** | **1^st^**  **week** | **2^nd^ week** | **4^th^ week** | **6^th^**  **week** | **8^th^**  **week** | **10^th^**  **week** | **12^th^ week** | **16^th^**  **week** | **20^th^**  **week** | **24^th^**  **week** | **28^th^**  **week** | **32^nd^**  **week** | **44^th^ week** | **56^th^**  **week** |
| **Hgb** | **12.5-17 g/dL** |  |  |  |  |  |  |  |  |  |  |  |  |  |  |  |
| **WBC** | **4.0-10.5x 10^3^** |  |  |  |  |  |  |  |  |  |  |  |  |  |  |  |
| **Neutrophils** | **1.8-7.8 x 10^3^** |  |  |  |  |  |  |  |  |  |  |  |  |  |  |  |
| **ESR** | **0-20 mm1^st^ hr** |  |  |  |  |  |  |  |  |  |  |  |  |  |  |  |
| **Urine HCG** | **Negative** |  |  |  |  |  |  |  |  |  |  |  |  |  |  |  |
| **Platelet** | **140-400x 10^3^** |  |  |  |  |  |  |  |  |  |  |  |  |  |  |  |
| **Sputum AFB** | **Negative** |  |  |  |  |  |  |  |  |  |  |  |  |  |  |  |
| **FBS** | **110mgs/dL** |  |  |  |  |  |  |  |  |  |  |  |  |  |  |  |
| **AST** | **0-60 iu/L** |  |  |  |  |  |  |  |  |  |  |  |  |  |  |  |
| **ALT** | **0-50 iu/L** |  |  |  |  |  |  |  |  |  |  |  |  |  |  |  |
| **ALP** | **0-483 iu/L** |  |  |  |  |  |  |  |  |  |  |  |  |  |  |  |
| **Bil (T)** | **0.1-1.2mg/dL** |  |  |  |  |  |  |  |  |  |  |  |  |  |  |  |
| **Bil (D)** | **0-0.4 mg/dL** |  |  |  |  |  |  |  |  |  |  |  |  |  |  |  |
| **T.Protein** | **6-8 g/dL** |  |  |  |  |  |  |  |  |  |  |  |  |  |  |  |
| **Albumin** | **3.5-5.5 g/dL** |  |  |  |  |  |  |  |  |  |  |  |  |  |  |  |
| **Urea** | **5-18 mg/dL** |  |  |  |  |  |  |  |  |  |  |  |  |  |  |  |
| **Creatinine** | **0.6-1.1mg/dL** |  |  |  |  |  |  |  |  |  |  |  |  |  |  |  |
| **HBsAg** | **Negative** |  |  |  |  |  |  |  |  |  |  |  |  |  |  |  |
| **HCV Ab** | **Negative** |  |  |  |  |  |  |  |  |  |  |  |  |  |  |  |
| **VDRL** | **Non reactive** |  |  |  |  |  |  |  |  |  |  |  |  |  |  |  |
| **CD4** | **500-1400** |  |  |  |  |  |  |  |  |  |  |  |  |  |  |  |
| **HIV RNA** | **LDL** |  |  |  |  |  |  |  |  |  |  |  |  |  |  |  |
| **CXR (PA)** | **Normal** |  | **Base line CXR (PA) is required for all cases enrolled in the study. It is repeated only when required.** | | | | | | | | | | | | | |
| **TST** |  |  | **TST will be repeated only when IRIS is suspected or diagnosed.** | | | | | | | | | | | | | |
| **Abd. Sono*** | **Normal** |  | **Abdominal sonography will be done only if it is required.** | | | | | | | | | | | | | |
| **Note: Shaded zones the specific lab tests will not be done unless indicated. RFT will be repeated only if required.** | | | | | | | | | | | | | | | | |

**HAART at 8 weeks**

**Scheduled Follow up visits for Clinical examination**

**(Note: The following forms will be filled. Annex 1 if any Drug Adverse Effect is diagnosed,**

**Annex 2 if DIH is diagnosed and Annex 3 to investigate Neuropsychiatry changes related with EFV.)**

| **Visit No** | **Date** | **History** | **P/exam** | **Remark** | | |
| --- | --- | --- | --- | --- | --- | --- |
| **Week 2** |  | **Symptoms today**  _______________________  _______________________  _______________________  _______________________  _______________________  _______________________  _______________________  _______________________  _______________________  _______________________  _______________________  **Other Diagnoses**  (between last visit and index visit)  _______________________  _______________________  _______________________  **Weight = ______ kg**  **Temp: ______°C**  **BP: ______/_____mmHg**  **PR:_________b/min**  **RR: ________breaths/min** | 1) Enlarged Lymph nodes   No  Yes  Cervical _______  Axillary _______  Inguinal _______  2) Skin and mucous membrane   Normal  Abnormal  _______________________  3) Lungs   Normal  Abnormal  _______________________  4) Abdomen   Normal  Abnormal  _______________________  5) Cardiovascular   Normal  Abnormal  _______________________  6) Neurology   Normal  Abnormal  _______________________  **Clinical Summary**  Karnofsky score ________  WHO HIV stage _________  **Working diagnosis**  1) _____________________  2) _____________________  3) _____________________  4) _____________________  **Treatment offered today**  1) _____________________  2) _____________________  3) _____________________  4) _____________________  5) _____________________  6) _____________________ | **Variable** | **No** | **Yes** |
|  |  |  |  | **New ADI or OI**  **(AIDS Defining Illness)** |  |  |
|  |  |  |  | **Specify the new ADI or OI=** | | |
|  |  |  |  | **Duration of HAART**  **before the new ADI** | | |
|  |  |  |  | **DIH**  **Yes response, fill in Annex 2** |  |  |
|  |  |  |  | **Neuropsychiatry changes related with EFV (Fill in the form)** |  |  |
|  |  |  |  | **Skin rash**  **(ACTG grade)** |  |  |
|  |  |  |  | **Anemia**  **(ACTG grade)** |  |  |
|  |  |  |  | **Peripheral neuropathy**  **(ACTG grade 1-4)** |  |  |
|  |  |  |  | **TB IRIS** |  |  |
|  |  |  |  | **Missed any ARV dose the past 3 days** |  |  |
|  |  |  |  | **Any missed anti TB drug/s**  **(Refer to the TB treatment card)** |  |  |
|  |  |  |  | **Over all patient status** | | |
|  |  |  |  | **Same** |  |  |
|  |  |  |  | **Worsened** |  |  |
|  |  |  |  | **Improved** |  |  |
|  |  |  |  | **Died** |  |  |
|  |  |  |  |  |  |  |

**Scheduled Follow up visits for Clinical examination**

**(Note: The following forms will be filled. Annex 1 if any Drug Adverse Effect is diagnosed,**

**Annex 2 if DIH is diagnosed and Annex 3 to investigate Neuropsychiatry changes related with EFV.)**

| **Visit No** | **Date** | **History** | **P/exam** | **Remark** | | |
| --- | --- | --- | --- | --- | --- | --- |
| **Week 4** |  | **Symptoms today**  _______________________  _______________________  _______________________  _______________________  _______________________  _______________________  _______________________  _______________________  _______________________  _______________________  _______________________  **Other Diagnoses**  (between last visit and index visit)  _______________________  _______________________  _______________________  **Weight = ______ kg**  **Temp: ______°C**  **BP: ______/_____mmHg**  **PR:_________b/min**  **RR: ________breaths/min** | 1) Enlarged Lymph nodes   No  Yes  Cervical _______  Axillary _______  Inguinal _______  2) Skin and mucous membrane   Normal  Abnormal  _______________________  3) Lungs   Normal  Abnormal  _______________________  4) Abdomen   Normal  Abnormal  _______________________  5) Cardiovascular   Normal  Abnormal  _______________________  6) Neurology   Normal  Abnormal  _______________________  **Clinical Summary**  Karnofsky score ________  WHO HIV stage _________  **Working diagnosis**  1) _____________________  2) _____________________  3) _____________________  4) _____________________  **Treatment offered today**  1) _____________________  2) _____________________  3) _____________________  4) _____________________  5) _____________________  6) _____________________ | **Variable** | **No** | **Yes** |
|  |  |  |  | **New ADI or OI**  **(AIDS Defining Illness)** |  |  |
|  |  |  |  | **Specify the new ADI or OI=** | | |
|  |  |  |  | **Duration of HAART**  **before the new ADI** | | |
|  |  |  |  | **DIH**  **Yes response, fill in Annex 2** |  |  |
|  |  |  |  | **Neuropsychiatry changes related with EFV (Fill in the form)** |  |  |
|  |  |  |  | **Skin rash**  **(ACTG grade)** |  |  |
|  |  |  |  | **Anemia**  **(ACTG grade)** |  |  |
|  |  |  |  | **Peripheral neuropathy**  **(ACTG grade 1-4)** |  |  |
|  |  |  |  | **TB IRIS** |  |  |
|  |  |  |  | **Missed any ARV dose the past 3 days** |  |  |
|  |  |  |  | **Any missed anti TB drug/s**  **(Refer to the TB treatment card)** |  |  |
|  |  |  |  | **Over all patient status** | | |
|  |  |  |  | **Same** |  |  |
|  |  |  |  | **Worsened** |  |  |
|  |  |  |  | **Improved** |  |  |
|  |  |  |  | **Died** |  |  |
|  |  |  |  |  |  |  |

**Scheduled Follow up visits for Clinical examination**

**(Note: The following forms will be filled. Annex 1 if any Drug Adverse Effect is diagnosed,**

**Annex 2 if DIH is diagnosed and Annex 3 to investigate Neuropsychiatry changes related with EFV.)**

| **Visit No** | **Date** | **History** | **P/exam** | **Remark** | | |
| --- | --- | --- | --- | --- | --- | --- |
| **Week 8** |  | **Symptoms today**  _______________________  _______________________  _______________________  _______________________  _______________________  _______________________  _______________________  _______________________  _______________________  _______________________  _______________________  **Other Diagnoses**  (between last visit and index visit)  _______________________  _______________________  _______________________  **Weight = ______ kg**  **Temp: ______°C**  **BP: ______/_____mmHg**  **PR:_________b/min**  **RR: ________breaths/min** | 1) Enlarged Lymph nodes   No  Yes  Cervical _______  Axillary _______  Inguinal _______  2) Skin and mucous membrane   Normal  Abnormal  _______________________  3) Lungs   Normal  Abnormal  _______________________  4) Abdomen   Normal  Abnormal  _______________________  5) Cardiovascular   Normal  Abnormal  _______________________  6) Neurology   Normal  Abnormal  _______________________  **Clinical Summary**  Karnofsky score ________  WHO HIV stage _________  **Working diagnosis**  1) _____________________  2) _____________________  3) _____________________  4) _____________________  **Treatment offered today**  1) _____________________  2) _____________________  3) _____________________  4) _____________________  5) _____________________  6) _____________________ | **Variable** | **No** | **Yes** |
|  |  |  |  | **New ADI or OI**  **(AIDS Defining Illness)** |  |  |
|  |  |  |  | **Specify the new ADI or OI=** | | |
|  |  |  |  | **Duration of HAART**  **before the new ADI** | | |
|  |  |  |  | **DIH**  **Yes response, fill in Annex 2** |  |  |
|  |  |  |  | **Neuropsychiatry changes related with EFV (Fill in the form)** |  |  |
|  |  |  |  | **Skin rash**  **(ACTG grade)** |  |  |
|  |  |  |  | **Anemia**  **(ACTG grade)** |  |  |
|  |  |  |  | **Peripheral neuropathy**  **(ACTG grade 1-4)** |  |  |
|  |  |  |  | **TB IRIS** |  |  |
|  |  |  |  | **Missed any ARV dose the past 3 days** |  |  |
|  |  |  |  | **Any missed anti TB drug/s**  **(Refer to the TB treatment card)** |  |  |
|  |  |  |  | **Over all patient status** | | |
|  |  |  |  | **Same** |  |  |
|  |  |  |  | **Worsened** |  |  |
|  |  |  |  | **Improved** |  |  |
|  |  |  |  | **Died** |  |  |

| **Visit No** | **Date** | **History** | **P/exam** | **Remark** | | |
| --- | --- | --- | --- | --- | --- | --- |
| **Week 12** |  | **Symptoms today**  _______________________  _______________________  _______________________  _______________________  _______________________  _______________________  _______________________  _______________________  _______________________  _______________________  _______________________  **Other Diagnoses**  (between last visit and index visit)  _______________________  _______________________  _______________________  **Weight = ______ kg**  **Temp: ______°C**  **BP: ______/_____mmHg**  **PR:_________b/min**  **RR: ________breaths/min** | 1) Enlarged Lymph nodes   No  Yes  Cervical _______  Axillary _______  Inguinal _______  2) Skin and mucous membrane   Normal  Abnormal  _______________________  3) Lungs   Normal  Abnormal  _______________________  4) Abdomen   Normal  Abnormal  _______________________  5) Cardiovascular   Normal  Abnormal  _______________________  6) Neurology   Normal  Abnormal  _______________________  **Clinical Summary**  Karnofsky score ________  WHO HIV stage _________  **Working diagnosis**  1) _____________________  2) _____________________  3) _____________________  4) _____________________  **Treatment offered today**  1) _____________________  2) _____________________  3) _____________________  4) _____________________  5) _____________________  6) _____________________ | **Variable** | **No** | **Yes** |
|  |  |  |  | **New ADI or OI**  **(AIDS Defining Illness)** |  |  |
|  |  |  |  | **Specify the new ADI or OI=** | | |
|  |  |  |  | **Duration of HAART**  **before the new ADI** | | |
|  |  |  |  | **DIH**  **Yes response, fill in Annex 2** |  |  |
|  |  |  |  | **Neuropsychiatry changes related with EFV (Fill in the form)** |  |  |
|  |  |  |  | **Skin rash**  **(ACTG grade)** |  |  |
|  |  |  |  | **Anemia**  **(ACTG grade)** |  |  |
|  |  |  |  | **Peripheral neuropathy**  **(ACTG grade 1-4)** |  |  |
|  |  |  |  | **TB IRIS** |  |  |
|  |  |  |  | **Missed any ARV dose the past 3 days** |  |  |
|  |  |  |  | **Any missed anti TB drug/s**  **(Refer to the TB treatment card)** |  |  |
|  |  |  |  | **Over all patient status** | | |
|  |  |  |  | **Same** |  |  |
|  |  |  |  | **Worsened** |  |  |
|  |  |  |  | **Improved** |  |  |
|  |  |  |  | **Died** |  |  |

**Scheduled Follow up visits for Clinical examination**

**(Note: The following forms will be filled. Annex 1 if any Drug Adverse Effect is diagnosed,**

**Annex 2 if DIH is diagnosed and Annex 3 to investigate Neuropsychiatry changes related with EFV.)**

**Scheduled Follow up visits for Clinical examination**

**Note: The following forms will be filled. Annex 1 if any Drug Adverse Effect is diagnosed, Annex 2 if DIH is diagnosed and for every patient at all scheduled visits fill in the format to investigate Neuropsychiatry changes related with EFV.)**

| **Visit No** | **Date** | **History** | **P/exam** | **Remark** | | |
| --- | --- | --- | --- | --- | --- | --- |
| **Week 20** |  | **Symptoms today**  _______________________  _______________________  _______________________  _______________________  _______________________  _______________________  _______________________  _______________________  _______________________  _______________________  _______________________  **Other Diagnoses**  (between last visit and index visit)  _______________________  _______________________  _______________________  **Weight = ______ kg**  **Temp: ______°C**  **BP: ______/_____mmHg**  **PR:_________b/min**  **RR: ________breaths/min** | 1) Enlarged Lymph nodes   No  Yes  Cervical _______  Axillary _______  Inguinal _______  2) Skin and mucous membrane   Normal  Abnormal  _______________________  3) Lungs   Normal  Abnormal  _______________________  4) Abdomen   Normal  Abnormal  _______________________  5) Cardiovascular   Normal  Abnormal  _______________________  6) Neurology   Normal  Abnormal  _______________________  **Clinical Summary**  Karnofsky score ________  WHO HIV stage _________  **Working diagnosis**  1) _____________________  2) _____________________  3) _____________________  4) _____________________  **Treatment offered today**  1) _____________________  2) _____________________  3) _____________________  4) _____________________  5) _____________________  6) _____________________ | **Variable** | **No** | **Yes** |
|  |  |  |  | **New ADI or OI**  **(AIDS Defining Illness)** |  |  |
|  |  |  |  | **Specify the new ADI or OI=** | | |
|  |  |  |  | **Duration of HAART**  **before the new ADI** | | |
|  |  |  |  | **DIH**  **Yes response, fill in Annex 2** |  |  |
|  |  |  |  | **Neuropsychiatry changes related with EFV (Fill Annex 3)** |  |  |
|  |  |  |  | **Skin rash**  **(ACTG grade)** |  |  |
|  |  |  |  | **Anemia**  **(ACTG grade)** |  |  |
|  |  |  |  | **Peripheral neuropathy**  **(ACTG grade 1-4)** |  |  |
|  |  |  |  | **TB IRIS** |  |  |
|  |  |  |  | **Missed any ARV dose the past 3 days** |  |  |
|  |  |  |  | **Any missed anti TB drug/s**  **(Refer to the TB treatment card)** |  |  |
|  |  |  |  | **Over all patient status** | | |
|  |  |  |  | **Same** |  |  |
|  |  |  |  | **Worsened** |  |  |
|  |  |  |  | **Improved** |  |  |
|  |  |  |  | **Died** |  |  |
|  |  |  |  |  |  |  |

|  |  |  |  |  |
| --- | --- | --- | --- | --- |

**Scheduled Follow up visits for Clinical examination**

**(Note: The following forms will be filled. Annex 1 if any Drug Adverse Effect is diagnosed,**

**Annex 2 if DIH is diagnosed and Annex 3 to investigate Neuropsychiatry changes related with EFV**

**are diagnosed.)**

| **Visit No** | **Date** | **History** | **P/exam** | **Remark** | | |
| --- | --- | --- | --- | --- | --- | --- |
| **Week 24** |  | **Symptoms today**  _______________________  _______________________  _______________________  _______________________  _______________________  _______________________  _______________________  _______________________  _______________________  _______________________  _______________________  **Other Diagnoses**  (between last visit and index visit)  _______________________  _______________________  _______________________  **Weight = ______ kg**  **Temp: ______°C**  **BP: ______/_____mmHg**  **PR:_________b/min**  **RR: ________breaths/min** | 1) Enlarged Lymph nodes   No  Yes  Cervical _______  Axillary _______  Inguinal _______  2) Skin and mucous membrane   Normal  Abnormal  _______________________  3) Lungs   Normal  Abnormal  _______________________  4) Abdomen   Normal  Abnormal  _______________________  5) Cardiovascular   Normal  Abnormal  _______________________  6) Neurology   Normal  Abnormal  _______________________  **Clinical Summary**  Karnofsky score ________  WHO HIV stage _________  **Working diagnosis**  1) _____________________  2) _____________________  3) _____________________  4) _____________________  **Treatment offered today**  1) _____________________  2) _____________________  3) _____________________  4) _____________________  5) _____________________  6) _____________________ | **Variable** | **No** | **Yes** |
|  |  |  |  | **New ADI or OI**  **(AIDS Defining Illness)** |  |  |
|  |  |  |  | **Specify the new ADI or OI=** | | |
|  |  |  |  | **Duration of HAART**  **before the new ADI** | | |
|  |  |  |  | **DIH**  **Yes response, fill in Annex 2** |  |  |
|  |  |  |  | **Neuropsychiatry changes related with EFV (Fill Annex 3)** |  |  |
|  |  |  |  | **Skin rash**  **(ACTG grade)** |  |  |
|  |  |  |  | **Anemia**  **(ACTG grade)** |  |  |
|  |  |  |  | **Peripheral neuropathy**  **(ACTG grade 1-4)** |  |  |
|  |  |  |  | **TB IRIS** |  |  |
|  |  |  |  | **Missed any ARV dose the past 3 days** |  |  |
|  |  |  |  | **Any missed anti TB drugs**  **(Refer to the TB treatment card)** |  |  |
|  |  |  |  | **Over all patient status** | | |
|  |  |  |  | **Same** |  |  |
|  |  |  |  | **Worsened** |  |  |
|  |  |  |  | **Improved** |  |  |
|  |  |  |  | **Died** |  |  |
|  |  |  |  |  |  |  |

**Scheduled Follow up visits for Clinical examination**

**(Note: The following forms will be filled. Annex 1 if any Drug Adverse Effect is diagnosed,**

**Annex 2 if DIH is diagnosed and Annex 3 to investigate Neuropsychiatry changes related with EFV**

**are diagnosed.)**

| **Visit No** | **Date** | **History** | **P/exam** | **Remark** | | |
| --- | --- | --- | --- | --- | --- | --- |
| **Week 28** |  | **Symptoms today**  _______________________  _______________________  _______________________  _______________________  _______________________  _______________________  _______________________  _______________________  _______________________  _______________________  _______________________  **Other Diagnoses**  (between last visit and index visit)  _______________________  _______________________  _______________________  **Weight = ______ kg**  **Temp: ______°C**  **BP: ______/_____mmHg**  **PR:_________b/min**  **RR: ________breaths/min** | 1) Enlarged Lymph nodes   No  Yes  Cervical _______  Axillary _______  Inguinal _______  2) Skin and mucous membrane   Normal  Abnormal  _______________________  3) Lungs   Normal  Abnormal  _______________________  4) Abdomen   Normal  Abnormal  _______________________  5) Cardiovascular   Normal  Abnormal  _______________________  6) Neurology   Normal  Abnormal  _______________________  **Clinical Summary**  Karnofsky score ________  WHO HIV stage _________  **Working diagnosis**  1) _____________________  2) _____________________  3) _____________________  4) _____________________  **Treatment offered today**  1) _____________________  2) _____________________  3) _____________________  4) _____________________  5) _____________________  6) _____________________ | **Variable** | **No** | **Yes** |
|  |  |  |  | **New ADI or OI**  **(AIDS Defining Illness)** |  |  |
|  |  |  |  | **Specify the new ADI or OI=** | | |
|  |  |  |  | **Duration of HAART**  **before the new ADI** | | |
|  |  |  |  | **DIH**  **Yes response, fill in Annex 2** |  |  |
|  |  |  |  | **Neuropsychiatry changes related with EFV (Fill Annex 3)** |  |  |
|  |  |  |  | **Skin rash**  **(ACTG grade)** |  |  |
|  |  |  |  | **Anemia**  **(ACTG grade)** |  |  |
|  |  |  |  | **Peripheral neuropathy**  **(ACTG grade 1-4)** |  |  |
|  |  |  |  | **TB IRIS** |  |  |
|  |  |  |  | **Missed any ARV dose the past 3 days** |  |  |
|  |  |  |  | **Any missed anti TB drug/s**  **(Refer to the TB treatment card)** |  |  |
|  |  |  |  | **Over all patient status** | | |
|  |  |  |  | **Same** |  |  |
|  |  |  |  | **Worsened** |  |  |
|  |  |  |  | **Improved** |  |  |
|  |  |  |  | **Died** |  |  |
|  |  |  |  |  |  |  |

**Scheduled Follow up visits for Clinical examination**

**(Note: The following forms will be filled. Annex 1 if any Drug Adverse Effect is diagnosed,**

**Annex 2 if DIH is diagnosed and Annex 3 to investigate Neuropsychiatry changes related with EFV**

**are diagnosed.)**

| **Visit No** | **Date** | **History** | **P/exam** | **Remark** | | |
| --- | --- | --- | --- | --- | --- | --- |
| **Week 48 or 52** |  | **Symptoms today**  _______________________  _______________________  _______________________  _______________________  _______________________  _______________________  _______________________  _______________________  _______________________  _______________________  _______________________  **Other Diagnoses**  (between last visit and index visit)  _______________________  _______________________  _______________________  **Weight = ______ kg**  **Temp: ______°C**  **BP: ______/_____mmHg**  **PR:_________b/min**  **RR: ________breaths/min** | 1) Enlarged Lymph nodes   No  Yes  Cervical _______  Axillary _______  Inguinal _______  2) Skin and mucous membrane   Normal  Abnormal  _______________________  3) Lungs   Normal  Abnormal  _______________________  4) Abdomen   Normal  Abnormal  _______________________  5) Cardiovascular   Normal  Abnormal  _______________________  6) Neurology   Normal  Abnormal  _______________________  **Clinical Summary**  Karnofsky score ________  WHO HIV stage _________  **Working diagnosis**  1) _____________________  2) _____________________  3) _____________________  4) _____________________  **Treatment offered today**  1) _____________________  2) _____________________  3) _____________________  4) _____________________  5) _____________________  6) _____________________ | **Variable** | **No** | **Yes** |
|  |  |  |  | **New ADI or OI**  **(AIDS Defining Illness)** |  |  |
|  |  |  |  | **Specify the new ADI or OI=** | | |
|  |  |  |  | **Duration of HAART**  **before the new ADI** | | |
|  |  |  |  | **DIH**  **Yes response, fill in Annex 2** |  |  |
|  |  |  |  | **Neuropsychiatry changes related with EFV (Fill Annex 3)** |  |  |
|  |  |  |  | **Skin rash**  **(ACTG grade)** |  |  |
|  |  |  |  | **Anemia**  **(ACTG grade)** |  |  |
|  |  |  |  | **Peripheral neuropathy**  **(ACTG grade 1-4)** |  |  |
|  |  |  |  | **TB IRIS** |  |  |
|  |  |  |  | **Missed any ARV dose the past 3 days** |  |  |
|  |  |  |  | **Any missed anti TB drug/s**  **(Refer to the TB treatment card)** |  |  |
|  |  |  |  | **Over all patient status** | | |
|  |  |  |  | **Same** |  |  |
|  |  |  |  | **Worsened** |  |  |
|  |  |  |  | **Improved** |  |  |
|  |  |  |  | **Died** |  |  |
|  |  |  |  |  |  |  |

**Annex 1**

**Reporting Serious adverse events (Grade 3 & 4 events)**

**Patient demographics**

Sex  Male  Female

Date of birth __ __ __ __ __ __

dd mm yyyy

Height_____________cm Weight_____________kg

**Specify the adverse event**

Event in medical terms__________________________________________

List suspected drug/s: __________________________________________________

Indicate the daily dose of the drug/s at the onset of event

_____________________________________________________

_____________________________________________________

_____________________________________________________

Therapy dates from __ __ __ __ __ __

dd mm yyyy

Therapy duration until onset ______________

hh dd mm

End date __ __ __ __ __ __ _ __

dd mm yyyy

Intensity/ Severity  Grade 1  Grade 2  Grade 3  Grade 4

Did the event abate after stopping product?  No  Yes  Not available

##### Severity criteria:

Transient or mild discomfort; no limitation of activity; no medical intervention/ therapy require (Grade 1)

Mild to moderate limitation of activity; some assistance may be needed; no or minimal medical intervention/ therapy required (Grade 2).

Marked limitation in activity; some assistance usually required; medical intervention/ therapy required; hospitalization possible (Grade 3)

Extreme limitation of activity; significant assistance required; significant medical intervention/therapy required; hospitalization or hospice care (Grade 4)

Death, date: ________________ Autopsy  Yes  No

**Action taken by the investigator:**

None

All antiretroviral drugs discontinued.

Anti TB drugs discontinued

Dose of d4T changed from 40 mgs BID to 30 mgs BID

d4T shifted to AZT.

AZT shifted to d4T.

.

New drug therapy added.

Concomitant medication discontinued.

Hospital admission

##### Event outcome

Completely recovered on _______________

dd mm yyyy

Recovered with sequel  Condition improving

Condition still unchanged  Condition deteriorated

Unknown  Death (specify date) ___________

**Causality assessment by the investigator to the drug/s**

Not related  Probable

Unlikely  Most probable

Possible  Insufficient data to assess

**Concomitant medication –identify all medications taken at the time of SAE**

| Drug name | Single, dose/unit  **(Eg. 500mg)** | **Frequency of this dose** | **Start**  **Date/time** | **End**  **Date/time** | Comments |
| --- | --- | --- | --- | --- | --- |
|  |  |  |  |  |  |
|  |  |  |  |  |  |
|  |  |  |  |  |  |
|  |  |  |  |  |  |
|  |  |  |  |  |  |
|  |  |  |  |  |  |

Laboratory data –identify any test result relevant to SAE

| Test | Date | Result |
| --- | --- | --- |
|  |  |  |
|  |  |  |
|  |  |  |
|  |  |  |
|  |  |  |
|  |  |  |

## Narrative and additional information:

**Annex 2**

**Monitoring hepatotoxicity**

1. Number of days the patient received anti TB before the diagnosis of DIH was made _______

2. Number of days patient received HAART before DIH was diagnosed? ________________

3. Grade DIH according to ACTG

(Use the ALT level for grading purposes)

| **Visit No** | **Date** | **Grade 1**  **1.25-2.5x**  **ULN** | **Grade 2**  **>2.5-5.0x ULN** | **Grade 3**  **> 5.0-10.0 x ULN** | **Grade 4**  **>10x ULN** | **Remark** |
| --- | --- | --- | --- | --- | --- | --- |
|  |  |  |  |  |  |  |
|  |  |  |  |  |  |  |
|  |  |  |  |  |  |  |
|  |  |  |  |  |  |  |
|  |  |  |  |  |  |  |
|  |  |  |  |  |  |  |
|  |  |  |  |  |  |  |
|  |  |  |  |  |  |  |
|  |  |  |  |  |  |  |
|  |  |  |  |  |  |  |
|  |  |  |  |  |  |  |

3. Have the anti TB drugs been totally discontinued? No Yes (specify date)

_____________________________________________________________________________

4. Restarting anti TB drugs:

| **Restarted anti TB after total discontinuation** | **Date** | **INH 50 mgs** | **INH 100 mgs** | **INH 200 mgs+RIF 300 mgs** | **RHZ 1 tab + RH 2 tabs** | **RHZ**  **2 tabs +**  **RH 1 tab** | **RHZE** | **Remark** |
| --- | --- | --- | --- | --- | --- | --- | --- | --- |
| **First** |  |  |  |  |  |  |  |  |
| **Second** |  |  |  |  |  |  |  |  |
| **Third** |  |  |  |  |  |  |  |  |
| **Fourth** |  |  |  |  |  |  |  |  |
| **Fifth** |  |  |  |  |  |  |  |  |
| **All** |  |  |  |  |  |  |  |  |

**Note: RHZ (R= 150 mgs, H= 75 mgs, Z= 400 mgs), RH (R= 150 mgs, H= 75 mgs),**

**RHZE (R= 150 mgs, H= 75 mgs, Z= 400 mgs, E= 275mgs)**

5. Did the level of the aminotransferases warrant discontinuation of HAART? No Yes

6. After how many days of total discontinuation was HAART reinitiated? (Specify the date)

_______________________________________________________________________________

7. Specify the 2^nd^ line regimen used to reinitiate HAART?

_________________________________________________________________________________

8. Final outcome of the DIH Recovered Death Do not know

9. If the final outcome is death, indicate the cause of death.

Hepatic Non-hepatic

Further remarks on the course of DIH

| **Date** | **Complaints** | **P/exam** | **Remark** |
| --- | --- | --- | --- |
|  |  |  |  |

**Annex**

**WHO Clinical staging of HIV Disease in Adults and Adolescents**

**Karnofsky performance status score**

| **Value** | **Level of functional capacity** |
| --- | --- |
| 100 | Normal, no complaints, no evidence of disease |
| 90 | Able to carry on normal activity, minor signs or symptoms of disease |
| 80 | Normal activity with effort, some signs or symptoms of disease |
| 70 | Cares for self, unable to carry on normal activity or to do active work |
| 60 | Requires occasional assistance, but is able to care for most needs |
| 50 | Requires considerable assistance and frequent medical care |
| 40 | Disabled, requires special care and assistance |
| 30 | Severely disabled, hospitalization is indicated although death is not imminent |
| 20 | Hospitalization is necessary, very sick, active supportive treatment necessary |
| 10 | Moribund, fatal processes progressing rapidly |
| 0 | Dead |

**ACTG staging classification for AIDS-related KS**

|  | **Good risk( all of the following)** | **Poor risk (any of the following)** |
| --- | --- | --- |
| Tumor, **T** | **T0**: Confined to skin and/or  lymph nodes and/or minimal oral disease (non-nodular KS confined to palate) | **T1**:Tumor-associated edema or ulceration  Extensive oral KS  Gastrointestinal KS  KS in other non-nodal viscera |
| Immune system, **I** | **I0**: CD4 cell count >200/µL* | **I1**: CD4 cell count <200/µL |
| Systemic illness, **S** | **S0**: No history of OI or thrush  No "B" symptoms  Karnofsky performance status >70 | **S1**: History of OI and/or thrush  "B" symptoms present  Karnofsky performance status <70  Other HIV-related illness  (e.g., neurological disease, lymphoma) |

**Annex**

**Severity Grading of Selected Clinical & Laboratory Toxicities**

**(To be filled at weeks 0, 2, 4 & 8)**

**Neuropsychiatry manifestations of Efavirenz**

Not present Mild Moderate Severe Extremely

severe

1. Vivid dreams?

0 1 2 3 4

2. Night mares?

0 1 2 3 4

3. How do you rate your

Sleep disturbance overall

the past one week? 0 1 2 3 4

4. Trouble staying awake

during the day time 0 1 2 3 4

5. Little interest or

pleasure in doing things 0 1 2 3 4

6. Feeling down hopeless

or depressed 0 1 2 3 4

7. Trouble falling or

staying asleep 0 1 2 3 4

8. Feeling tired or

having little energy 0 1 2 3 4

9. Poor appetite or

overeating 0 1 2 3 4

10. Feeling bad about

yourself 0 1 2 3 4

Not present Mild Moderate Severe Extremely

severe

11. Trouble concentrating on

things, such as reading

news paper or watching TV 0 1 2 3 4

12. Moving or speaking so

slowly that other people

could have noticed? 0 1 2 3 4

13. Thoughts that you would be

better off dead or hurting

yourself in some way 0 1 2 3 4

14. Anxious mood

(worries, anticipates worst)

0 1 2 3 4

15. Tension

(startles, cries easily,

Restless, trembling) 0 1 2 3 4

16. Fears

(dark, strangers,

being alone, unknown) 0 1 2 3 4

17. Felt light headed?

0 1 2 3 4

18. Headache?

0 1 2 3 4

19. Felt like I was going

to fall over? 0 1 2 3 4

Not present Mild Moderate Severe Extremely

severe

20. Felt like the room

was spinning? 0 1 2 3 4

21. Guilt feeling?

0 1 2 3 4

22. Hostility?

0 1 2 3 4

23. Elated mood?

0 1 2 3 4

24. Grandiosity?

0 1 2 3 4

25. Suspiciousness?

0 1 2 3 4

26. Hallucinations?

0 1 2 3 4

27. Unusual thought

Content? 0 1 2 3 4

28. Bizarre behavior

0 1 2 3 4

29. Self neglect?

0 1 2 3 4

30. Others?

0 1 2 3 4

##### Ethical consideration

This study is intended to identify the optimal time to initiate HAART in patients with CD4 counts less than 200 cells/mm^3^ after initial anti TB treatment. It will definitely improve the treatment outcome of patients with TB/HIV co-infection. All the study participants will be screened for HIV after pre-test counselling. Post-test counselling will also be given. They will be provided with the necessary information on the study benefits and risks. Every effort will be made to link HIV seropositives who require home based care or support with different social service providing organizations to get the best available care. Ethical approval will be sought both from Institutional and National ethics review committee.

**A. Anticipated benefits to the patients**

The final results of the study will definitely improve the treatment outcome of TB/HIV co-

infected patients in the Ethiopian setting. Individuals taking part in this clinical trial will

directly contribute to the result. At the study sites internists will follow the study

participants during the entire study period free of charge. Participants in this study will

benefit from knowing their HIV status, CD4 cell counts, viral load, early identification

and management of side effects associated with anti TB and/or HAART. Patients will be

able to withdraw from the study any time without having any repercussions on their

management. Expenses required for the treatment of opportunistic infections developing

during the study period including cost for laboratory studies will be covered from the

study expenses. Patients will not receive monetary compensation for being enrolled in

the study except remuneration for transportation as well as expenses related to the

diagnosis and management of TB/HIV co infection. However reasonable reimbursement

will be given for extra costs incurred due to participation in the study such as meals,

travel cost and for working time spent to participate in this trial. Directly Observed

Therapy -Short Course (DOTS) is the strategy we are going to follow with anti TB

therapy and the continuation phase of anti TB treatment will be completed with fixed

formulations of “RH” for four months. The study will provide fixed formulations of “RH”

and supplemental Vitamin B6 tablets.

**B. Description of potential risks to participants.**

All the benefits and potential risks of TB/HIV co treatment will be explained to the study participants. Increased mortality in the first month of treatment of HIV-infected tuberculosis patients seems largely attributable to tuberculosis itself, whereas other HIV-associated pathologies predominate thereafter. Although there is no doubt about the treatment benefit of both TB and HIV co-infections, clear definition of the optimal time to initiate HAART in these group of patients await results from controlled trials. The challenges during TB/HIV treatment include drug interactions, overlapping toxicities, non-adherence to treatment because of increased pill burden and IRIS. An important strategy we will be following to minimize drug-drug interactions as well as overlapping drug toxicities is to avoid starting both anti TB and HAART simultaneously. The long term consequence of the drug interactions between anti TB and HAART on the success of antiretroviral therapy is to be studied. The most important toxicities or adverse effects include gastro intestinal upset, drug induced hepatotoxicity and skin rash. Each of these will be managed accordingly. The amount of blood that is going to be drawn during the study period will be first explained to the study participants. The risk involved in this study is only minor bleeding from blood drawing site. The study does not require interruption of an ongoing anti TB or HAART regimens nor altering dosing interval.

**C. Methods to minimize risks**

Courses in standard of Good Clinical Practice (GCP) and Good Laboratory Practice (GLP) will be organized to the research staff at all trial sites. The courses will include basic principles of GCP and GLP. It will be provided with the assistance from our Karolinska Institute study collaborators. Compliance with good clinical and laboratory practice provides assurance that the right, safety and well being of the trial subjects is protected, and that the results of the clinical trial are credible. Standard Operating Procedures will be prepared for all routine methods to ensure that data are collected consistently and accurately. All the information including patient’s HIV status will be kept confidential. Participants’ medical records will only be accessible to the study group. It is emphasized on the consent form that the information about study participants will not be shared to others who are not directly involved in the care of study subjects.

The important challenges during treatment of TB/HIV co-infections will be drug interactions, overlapping toxicities, increased pill burden adversely affecting treatment adherence as well as TB IRIS. Patients will be explained about these treatment challenges and the fact that some of the risks can be encountered. There could be a risk of discontinuing anti TB and/or HAART if the adverse effects are found life threatening. The anti TB drugs will be temporarily discontinued if grade 3-hepatotoxicity develops with symptoms or grade 4- toxicity without symptoms. Indications to temporarily discontinue or substitute HAART will be Steven- Johnson’s syndrome, grade 4 hepatotoxicity, presumed lactic acidosis, peripheral neuropathy with intolerable symptoms, anaemia (Hgb < 8gms %) and pancytopenia. Reinstituting anti TB and switching the antiretroviral medications will be at discretion of the treating physicians.

Immune Reconstitution Inflammatory Syndrome (IRIS) which is one of the potential complications following the introduction of HAART will be closely followed. Steroids will be given if symptoms can not be controlled with NSAIDS. Minor gastro-intestinal upsets like dyspepsia, nausea and vomiting will be treated symptomatically.

An important step taken in this study is that the follow up visits are scheduled every week for the 1^st^ month of the study, every two weeks the second month and there after every 4 weeks until the study is completed. Emergency visits or consultations will be handled accordingly. Such close follow up visits will enable us to follow our patients closely and detect drug toxicities and undesirable effects of such treatment from becoming problems superimposed on the issues being acutely managed.

**D. Vulnerable populations**

The trial will not involve members of vulnerable populations, including pregnant women, prisoners or minors.

**E. Informed written / thumb sign consent**

All the study participants will be explained about the study and provided with written consent in Amharic (Ethiopian national language) to be written or thumb signed. Before signing the consent forms it will be read aloud to all potential participants. Interpreters will be used to assist those individuals who are not able to speak Amharic.

**F. Data and safety monitoring board**

Because this is a randomized clinical trial an independent data and safety monitoring board will be established before the clinical study is undertaken.

# Patient Information Sheet

###### Background: TB is the commonest opportunistic infection among HIV infected patients. TB/HIV co-treatment is recommended in patients with low CD4 counts. For patients with active TB in whom HIV infection is diagnosed and HAART is required the first priority is to initiate standard antituberculosis treatment. The high death rate in the first 2 months of tuberculosis treatment provides an argument for antiretroviral therapy to be started as soon as possible. However, challenges favouring delayed start include drug interactions, combined toxic effects, non-adherence to treatment and paradoxical worsening of tuberculosis. Clear definition of the best time to initiate antiretroviral treatment in patients with tuberculosis awaits results from controlled trials.

###### Objective: Aim of the present study is to investigate the optimal time to initiate HAART in patients with TB/HIV co infection presenting with active TB and at AIDS stage.

###### Significance of the study: Identification of the optimal time to initiate EFV-based HAART in patients taking anti TB treatment with underlying advanced immunosuppression helps to design better strategies and guidelines for HIV/TB co treatment.

###### Study site and period: The total period of the study is for 2 years but your participation requires one year.

###### Study Procedures: Newly diagnosed TB/HIV co infected patients with active TB and CD4 cell count< 200cells/mm^3^ will be recruited prospectively provided their consent is obtained. The study will have three arms. In all the cases initially anti TB treatment will be initiated according to the National treatment guideline and randomly the study subjects will be assigned to one of the three study arms. In the first arm EFV-based HAART will be initiated one week after the anti TB drugs are started and the second arm at 4 weeks while the last arm will initiate as soon as the first two months of intensive anti TB treatment are completed. You will be asked to give 1- 2 teaspoonful of blood (5-10ml) before initiation of HAART and anti TB medications and on the 1^st^ ,2^nd^, 4^th^, 6^th^, 8^th^ , 12^th^ 16^th^, 28^th^, 48^th^ or 52^nd^ weeks after you are enrolled in the study. This is in order to identify treatment adverse effects early and to monitor the safety and efficacy of both anti TB as well as antiretroviral therapy. Any drug including over the counter medication should be taken only with the knowledge and approval of the Principal Investigator.

**Benefits & risks**

The final results of the study will definitely improve the treatment outcome of TB/HIV

co- infected patients in Ethiopian setting. Individuals taking part in this clinical trial will

directly contribute to the result. At the study sites a consultant internist & a senior

Pulmonologist will follow the study participants during the entire study period free of

charge. Participants in this study will benefit from knowing their HIV status, CD4 cell

counts, viral load, early identification and management of side effects associated with

anti TB and/or HAART. Expenses required for the treatment of opportunistic infections

developing during the study period including cost for laboratory studies will be covered

from the study expenses. Patients will not receive monetary compensation for being

enrolled in the study except remuneration for transportation as well as expenses related

to the diagnosis and management of TB/HIV co infection. However reasonable

reimbursement will be given for extra costs incurred due to participation in the study

such as meals and travel costs spent to participate in this trial. Problems

in the first month of treatment of HIV-infected tuberculosis patients seem largely

attributable to tuberculosis itself, whereas other HIV-associated pathologies

predominate thereafter. Although there is no doubt about the treatment benefit of both

TB and HIV co-infections, clear definition of the optimal time to initiate HAART in these

group of patients await results from controlled trials. The challenges during TB/HIV

treatment include drug interactions, overlapping toxicities, non-adherence to treatment

because of increased pill burden and IRIS. The usual side effects from receiving the

anti TB and antiretroviral drugs may occur whether you participate in the study or not.

Any side effects should be reported to the Investigator. The most important toxicities or

adverse effects include gastro intestinal upset, drug induced hepatotoxicity and skin

rash. Each of these will be managed accordingly. The risk involved in this study is only

minor bleeding from blood drawing site. The study does not require interruption of an

ongoing anti-TB or HAART regimens or altering dosing interval.

**Sample storage:** Any sample collected will be stored at the research institutes until they are used up. The samples are kept coded and will be used for research purposes only. No information that discloses your identity will be released or published. You will not get payment for keeping the samples.

**Rights:** Participation in this study is entirely voluntary. Type(s) of drug treatment for the

participants is by no means different from those who are not participating in this study.

Initial voluntary HIV testing is required before participants are enrolled in the study.

Individuals above 18 years old and able to provide informed signed consent will be

involved in this study. You have the right to withhold information, refuse cooperation or

drop out of the study at any time you want with out any need to explain the reasons to

anyone, and that your actions will have no effect on the overall management of your

treatment. Patients will be able to withdraw from the study any time without having any

repercussions on their management. You have the right to ask and get clarifications at

any time.

**Confidentiality:** We will maintain the confidentiality of participants including your

personal identity and medical information. No information that discloses your identity will

be used in any reports about the study. In records that leave this study center will be

identified by a study code only. All information’s associated with this study will be kept

behind locked cabinets or in secure computer files. Your personal doctor and the

responsible researcher may inspect you medical records. However no records that

identify you will be allowed to leave the trial center.

###### Questions, Rights and Complaints: If you have any doubts, questions or desire further information with respect to the study, or if you experience any adverse effects, you may call Dr Wondwossen Amogne at 2519-1-1406179.

###### Consent form

###### I have been requested to participate in a research project entitled “Randomized clinical trial to determine safety and efficacy of immediate versus deferred initiation of HAART among treatment naïve HIV infected adults requiring treatment for smear positive Pulmonary and extrapulmonary TB.” This will require ascertaining my serostatus against HIV infection following pre and post test counselling.

###### I am informed that the 1^st^ two months of anti TB treatment will be according to the National Treatment Guideline of Ethiopia but the continuation phase of anti TB treatment will be with INH & Rifampicin which is co formulated as “HR” for 4 months. This formulation is used in the developed world and is known to be more successful than a combination of INH & Ethambutol (co formulated as EH). I have also realized that “HR” is preferred to “EH” by World Health Organization in the treatment of TB. I am explained that the anti TB drugs will be offered under direct observation the first two months and in blister packs for the next 4 months.

###### I have realized that there is no change in the standard of disease management. I know that I have the right to withhold information, refuse cooperation or drop-out of the study any time I want without any need to explain the reasons to anyone, and that my actions will have no effect on the overall management of my disease. I have been also informed that all the information I give to the interviewer will be kept confidential. I also know that I have the right to ask and get clarifications at any time. In case I have doubts or questions, I can contact Dr Wondwossen Amogne at 2519-1-1406179.

###### I have asked for clarification and I have received satisfactory responses in a language I can understand. I have been given enough time to respond. I finally confirm my agreement by putting my signature.

###### Signature of patient ______________________ Date _____________

###### Signature of the physician __________________ Date_________________

###### Signature of witness _____________________ Date ________________

###### Curriculum vitae of study participants

| **Lars Lindquist, MD, PhD, Professor of infectious diseases**  **Research interest: Tuberculosis and HIV interaction and pathogenesis of infectious diseases** |
| --- |
| **Bibliography or Publications: ref. and title**  **Lars Lindquist MD, PhD and professor at Karolinska Institute. Former head of the Clinic for infectious Diseases at the Karolinska University Hospital at Huddinge. He has been the main supervisor for 3 finished Swedish and one finished Lithuanian PhD. He is main supervisor for 2 ongoing PhD projects. He has presented 50 scientific articles in peer-reviewed journal. He is called as expert consultant in Infectious Diseases for the Stockholm County and member of several committees. He has for 3 years been the vice president in the Committee for Appointments for MD´s at the University Hospital at Karolinska, Huddinge. He is often called as referee by several scientific Journals. He has been the organizer and member of the Board of several international Conferences in the field of Infectious Disease. He has since 1980´s have had research collaboration with Ethiopia and is one of the organizers for the Swedish Ethiopian Course of Infectious Diseases in the Tropics to be held 2006.** |
|  |

| **Getachew Aderaye, MD, PhD Associate professor of Medicine & consultant chest physician, Department of Internal Medicine, Addis Ababa University.**  **Research interest: Clinical research on HIV & TB / Pneumocystis pneumonia (PCP).** |
| --- |
| **Dr. Getachew, apart from clinical service as an internist and pulmonologist, has interest in clinical research particularly around HIV and pulmonary opportunistic infections. He has served in several capacities in this field including ‘chairman of the organizing committee for the National AIDS case definition of Ethiopia (1993); chairman of the expert committee for the preparation of ‘Guidelines for the clinical management of HIV infection in adults & children in Ethiopia’; member of the organizing committee of ‘The first international conference on AIDS in Ethiopia’. Currently he serves as member of ‘The technical advisory board of ‘African AIDS Initiative International Inc.’ He is also a member of ‘TB research advisory committee (TRAC)’ of Ethiopia. As of 2003, he is serving as a member of the Developing Countries Co-ordinating Committee (DCCC), one of the constituencies of the EDCTP, representing the Horn of Africa on tuberculosis. Currently he is participating as a reviewer in the grant review process of the EDCTP. He is also serving as one of the expert members for the review of the guideline for the diagnosis and treatment of smear-negative tuberculosis, organized by WHO, Geneva. His main area of interest and publication is around HIV/ TB and PCP.** |
|  |
|  |
| **Eleni Aklillu, Bpharm, MSc, PhD**  **Research interest: Pharmacogenetics of drug metabolising enzyme, Pharmacokinetic, Pharmacokinetics and Pharmacodynamic of drug-drug interaction.** |
| | **Eleni Aklillu has received bachelor degree in Pharmacy from Addis Ababa University, MSc degree in Biochemistry (through a joint program between Karolinska University and Addis Ababa University) and PhD degree in Molecular genetics from Karolinska Institute, Stockholm. She has more than 10 years of experience in doing research regarding pharmacogenetic of drug metabolising enzymes with especial emphasis on Ethiopians. She has published more than 10 original scientific articles in peer reviewed international Journals. She has identified and characterized several polymorphisms in Cytochrome P450 enzymes with important implication in drug metabolism. She has investigated CYP2D6, CYP2C19, CYP2C9, CYP2B6, CYP1B1, CYP1A2, NAT2 etc in Ethiopian population. She has identified and published the unique nature of CYP2D6 polymorphism in Ethiopians i.e. the presence of CYP2D6 gene duplication and multi duplication carried by about 30 % Ethiopians (the frequency in other African and white population being <5%). She also identified novel polymorphisms in CYP1A2 (CYP1A2*1K) and in CYP1B1 (CYP1B1*7). Here are some of her publications.**  **Aklillu E, Øvrebø S, Botnen IV, Otter C, Ingelman-Sundberg M (2005) Characterization of common CYP1B1 variants with different capacity for benzo[*a*]pyrene-7,8-dihydrodiol epoxide formation from benzo[*a*]pyrene. *Cancer Research 65: 5105-5111.***  **Aklillu E, Carrillo JA, Makonnen E, Bertilsson L, Ingelman-Sundberg M (2003) Xanthine oxidase activity is influenced by environmental factors in Ethiopians. *Eur J Clin Pharmacol 59: 533-536.***  **Aklillu E, Carrillo JA, Makonnen E, Bertilsson L, Ingelman-Sundberg M (2003) Genetic polymorphism of CYP1A2 in Ethiopians affecting induction and expression. Characterization of novel haplotypes with SNPs in intron 1*. Mol Pharmacol 64: 659-669*.**  **Aklillu E. Pharmacogenetics of drug metabolizing enzymes with special emphasis on Ethiopians. PhD Thesis (**[**http://diss.kib.ki.se/2003/91-7349-460-7/thesis.pdf)**](http://diss.kib.ki.se/2003/91-7349-460-7/thesis.pdf)) **Karolinska Institutet, March (2003), KIB press, Stockholm.**  **Aklillu E, Oscarson M, Hidestrand M, Leidvik B, Otter C, Ingelman-Sundberg M (2002) Functional analysis of six different polymorphic CYP1B1 enzyme variants found in an Ethiopian population. *Mol Pharmacol 61: 586-594*.**  **Aklillu E, Herrlin K, Gustafsson LL, Bertilsson L, Ingelman-Sundberg M (2002) Evidence for environmental influence on CYP2D6-catalysed debrisoquine hydroxylation as demonstrated by phenotyping and genotyping of Ethiopians living in Ethiopia or in Sweden. *Pharmacogenetics 12: 375-383.***  **Aklillu E, Hidestrand M, Ingelman-Sundberg M, Malmebo S, Westlind A (2002) Recent Progress in Drug Metabolism Research. *Drug News Perspect*, 15: 528-534**  **Aklillu E, Persson I, Bertilsson L, Johansson I, Rodrigues F, Ingelman-Sundberg M (1996) Frequent distribution of ultrarapid metabolizers of debrisoquine in an Ethiopian population carrying duplicated and multiduplicated functional CYP2D6 alleles. *J Pharmacol Exp Ther 278: 441-446.*** | | --- | |
|  |
|  |
| **Wondwossen Amogne, MD, Assistant professor of Medicine, Department of Internal Medicine, Addis Ababa University.**  **Research interest: Clinical research on HIV & TB, ART** |
| **Wondwossen Amogne, MD, Assistant Professor in Internal Medicine, Addis Ababa University, Medical Faculty, working at the infectious diseases unit since 2005. He is involved both in the out patient ART clinic as well as in hospital care of patients with HIV/AIDS co infected with TB. He has been involved in the following research projects and published articles on Multifocal vertebral tuberculosis with involvement of the ribs (principal author) (EMJ 2002, vol 3, No 2); CNS toxoplasmosis in Ethiopian AIDS cases and Diabetic foot ulcer; Presentation and treatment experience in Addis Ababa University Main Teaching Hospital. The following are manuscripts are submitted to IUATLD for publication and are under review (co investigator). Antituberculous drug induced hepatotoxicity in HIV positive and negative patients and distribution and concordance of phenotypically and genotypically determined acetylation status on patients taking antituberculous drugs in Ethiopia. At present he is caring for nearly 1500 patients on antiretroviral therapy along with his colleagues in the ID unit. He is a member at the Ethiopian Medical Association HIV/AIDS advisory committee and was involved at several trainings on the effective use of antiretroviral therapy organized by the Ministry of Health, Ethiopia and Drug Administration and Control Authority, Ethiopia. He was trained as trainer for ART physicians organized by ITECH- Ethiopia.** |

##### 
